# An integrated single-cell and spatial transcriptomic atlas of thyroid cancer progression identifies prognostic fibroblast subpopulations

**DOI:** 10.1172/jci.insight.191990

**Published:** 2026-01-09

**Authors:** Matthew A. Loberg, George J. Xu, Sheau-Chiann Chen, Hua-Chang Chen, Claudia C. Wahoski, Kailey P. Caroland, Megan L. Tigue, Heather A. Hartmann, Jean-Nicolas Gallant, Courtney J. Phifer, Andres A. Ocampo, Dayle K. Wang, Reilly G. Fankhauser, Kirti A. Karunakaran, Chia-Chin Wu, Maxime Tarabichi, Sophia M. Shaddy, James L. Netterville, Sarah L. Rohde, Carmen C. Solórzano, Lindsay A. Bischoff, Naira Baregamian, Barbara A. Murphy, Jennifer H. Choe, Jennifer R. Wang, Eric C. Huang, Quanhu Sheng, Luciane T. Kagohara, Elizabeth M. Jaffee, Ryan H. Belcher, Ken S. Lau, Fei Ye, Ethan Lee, Vivian L. Weiss

**Affiliations:** 1 Department of Pathology, Microbiology, and Immunology;; 2 Department of Biostatistics;; 3 Department of Otolaryngology – Head and Neck Surgery; and; 4 Department of Medicine, Vanderbilt University Medical Center, Nashville, Tennessee, USA.; 5 Department of Genomic Medicine, The University of Texas MD Anderson Cancer Center, Houston, Texas, USA.; 6 The Francis Crick Institute, London, United Kingdom.; 7 Institute of Interdisciplinary Research (IRIBHM), Universite Libre de Bruxelles, Brussels, Belgium.; 8 Department of Laboratory Medicine and Pathology, University of Washington School of Medicine, Seattle, Washington, USA.; 9 Vanderbilt Ingram Cancer Center and; 10 Department of Surgery, Vanderbilt University Medical Center, Nashville, Tennessee, USA.; 11 Department of Head and Neck Surgery, The University of Texas MD Anderson Cancer Center, Houston, Texas, USA.; 12 Department of Oncology, Johns Hopkins University School of Medicine, Baltimore, Maryland, USA.; 13 Convergence Institute, Johns Hopkins University, Baltimore, Maryland, USA.; 14 Bloomberg-Kimmel Immunotherapy Institute, Johns Hopkins University School of Medicine, Baltimore, Maryland, USA.; 15 Vanderbilt Division of Pediatric Otolaryngology – Head and Neck Surgery, Monroe Carell Jr. Children’s Hospital at Vanderbilt, Nashville, Tennessee, USA.; 16 Department of Cell and Developmental Biology, Vanderbilt University, Nashville, Tennessee, USA.; 17 Epithelial Biology Center, Vanderbilt University Medical Center, Nashville, Tennessee, USA.; 18 Department of Pharmacology, Vanderbilt University, Nashville, Tennessee, USA.

**Keywords:** Genetics, Oncology, Cancer, Head and neck cancer, Thyroid disease

## Abstract

Although well-differentiated thyroid carcinoma (WDTC) is characterized by a robust treatment response, aggressive subtypes, such as anaplastic thyroid carcinoma (ATC), remain highly lethal. To understand thyroid cancer evolution in both children and adults, we analyzed single-cell transcriptomes of 423,733 cells from 81 samples and spatially resolved key tumor and microenvironment populations across 28 tumors with spatial transcriptomics, including rare and unique composite WDTC/ATC tumors and pediatric diffuse sclerosing thyroid carcinomas. Additionally, we identified gene signatures of stromal cell populations in 5 large thyroid cancer bulk RNA-sequencing cohorts. Through this multi-institutional effort, we defined a population of POSTN^+^ myofibroblast cancer-associated fibroblasts (myCAFs) that are intimately associated with invasive tumor cells and correlate with poor prognosis, lymph node metastasis, and disease progression in thyroid carcinoma. We also revealed a population of inflammatory CAFs that are distant to tumor cells and are found in the inflammatory stromal microenvironment of autoimmune thyroiditis. Together, our study provides spatial profiling of thyroid cancer evolution in samples with mixed WDTC/ATC histopathology and identifies a prognostic myCAF subtype with potential clinical utility in predicting aggressive disease in both children and adults.

## Abstract 2

Here, we present spatial transcriptomics data highlighting the stromal tumor-dynamics occurring across the spatial evolution of thyroid cancer from indolent to lethal disease.

## Introduction

In 1986, Harold Dvorak identified tumors as “wounds that do not heal” in recognition of the inflamed tumor microenvironment undergoing persistent extracellular matrix remodeling that resembles chronic wound healing (1). Fibroblasts, a central stromal component of wound healing, are highly plastic cells that become activated to remodel the extracellular matrix in response to tissue injury (2). Thus, in the chronic wound state of tumors, cancer-associated fibroblasts (CAFs) play a critical role in modulating the tumor microenvironment and tumor cell phenotypes.

Studies across solid tumors have implicated CAFs as supporting tumor progression and repressing antitumor immunity, making them promising therapeutic targets (3). However, attempts to broadly deplete CAFs in mouse models have resulted in aggressive tumor growth, highlighting the complex and tumor-restrictive properties of CAFs (4, 5). Clinical trials targeting CAFs or extracellular matrix have also had limited success (6). To improve CAF targeting and modulation strategies, multiple groups have sought to identify distinct tumor-promoting CAF subpopulations and functions. Single-cell sequencing efforts across tumor types have now defined numerous CAF subtypes (7–15). Broad CAF subtypes, including myofibroblast (myCAF), inflammatory (iCAF), and antigen-presenting, are commonly conserved across tumor types. However, the plastic and heterogenous CAF functionality recruited during tumor progression remains poorly defined, with subsets such as myCAFs serving critical roles in both matrix remodeling and immune modulation (12, 16). Additionally, several studies have identified perivascular populations as potential CAF precursors with CAF-like gene expression profiles (10, 11, 14). Despite identification of numerous CAF subtypes, CAFs remain an elusive clinical target. A better understanding of CAF phenotypes and plasticity within disease progression is needed to improve cancer therapeutics.

Well-differentiated thyroid cancers (WDTCs) are associated with excellent prognosis yet in rare instances clinically progress to anaplastic thyroid carcinoma (ATC) (17, 18), a carcinoma with an abysmal median survival of 3–5 months (19). Despite the rarity of WDTC clinical progression, ATCs often contain co-occurring, adjacent WDTC histologic regions, suggesting pathologic progression of WDTC to ATC (20–24). This spatial progression is accompanied by a robust accumulation of tumor stroma (25). The stark difference in tumor aggression and stromal infiltrate between WDTC and ATC, as well as their co-occurrence in ATC, make thyroid cancer an excellent model for tracking CAF phenotypes over spatial progression from curable to lethal disease. In our previous work, we developed a gene expression signature, the molecular aggression and prediction (MAP) score, that implicated fibroblasts as enriched in thyroid cancers at risk for disease recurrence, progression, or dedifferentiation to ATC (25). CAFs have previously been identified in thyroid cancer single-cell sequencing studies (26–29), yet a comprehensive profiling of CAF phenotypes over spatial progression is lacking.

In this study, we integrate single-cell RNA-sequencing data from 81 published thyroid cancer samples (26–32), generating a single-cell atlas to identify CAF subtypes across the landscape of tumor histology. Additionally, we perform spatial transcriptomics on 28 thyroid tumors to investigate CAF locations and interactions during thyroid cancer progression. Remarkably, we include 7 samples with co-occurring papillary (WDTC) and anaplastic histology, providing a distinct evaluation of the spatial progression of thyroid cancer. Finally, we map CAF subtypes to 5 large thyroid cancer bulk RNA-sequencing cohorts, demonstrating their potential prognostic role in metastasis and disease progression (31, 33). These data identify myCAFs as a potential biomarker for aggressive thyroid tumors and highlight their phenotype and tumor cell interactions during disease progression.

## Results

### Generation of thyroid cancer single-cell atlas.

**Figure 1:**
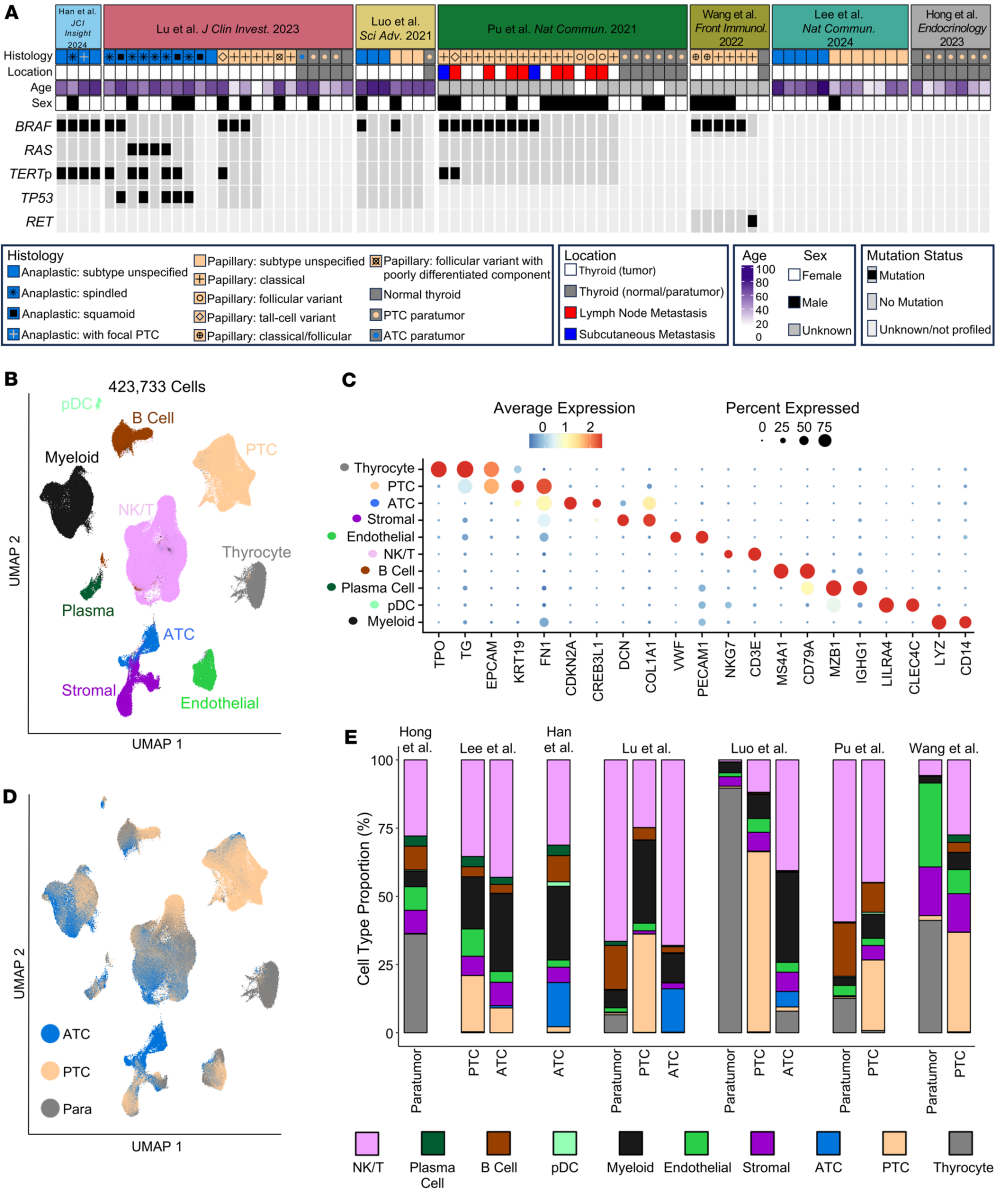
Integrated single-cell atlas of thyroid cancer progression. (**A**) Oncoplot for thyroid cancer publicly available single-cell RNA-sequencing samples: Luo et al. (26), Pu et al. (27), Lu et al. (28), Han et al. (29), Hong et al. (30), Lee et al. (31), Wang et al. (32). (**B**) Uniform manifold approximation and projection (UMAP) plot depicting the single-cell atlas labeled by broad cell type. (**C**) Scaled dot plot showing canonical markers for broad populations from **B**. (**D**) UMAP colored by tumor histology with broad groupings of ATC (blue), PTC (light orange), or paratumor/normal (Para, gray). (**E**) Bar plots showing overall broad cell type composition for each paper in the single-cell atlas split by tumor histology group. pDC, plasmacytoid dendritic cell; NK/T, natural killer/T cell.

We integrated published thyroid cancer single-cell RNA-sequencing samples from 7 papers with samples across the histologic and mutational landscape of thyroid cancer progression, including 20 normal thyroids/paratumor samples, 39 papillary thyroid cancers (PTCs; the most common WDTC), and 22 ATCs (Figure 1A and Supplemental Table 1; supplemental material available online with this article; https://doi.org/10.1172/jci.insight.191990DS1) (26–32). In total, 423,733 cells were integrated and clustered to identify broad cell populations (Figure 1B). Tumor, normal, and microenvironment populations expressing canonical marker genes were identified across samples from all 7 papers, including stromal cells with expression of fibroblast markers *DCN* and *COL1A1* (Figure 1, C–E, and Supplemental Figure 1, A–C). Most paratumor samples (15/17, 88%) had a low frequency (0%–7%) of PTC cells, consistent with their tumor-adjacent location. We further confirmed that thyrocyte and tumor cell populations had expected gene expression patterns by mapping previously developed thyroid differentiation score (TDS) and tumor scores (29, 33). TDS showed the highest enrichment in thyrocytes, a gradient in PTC cells, and no enrichment in ATC, consistent with dedifferentiation as tumors progress. *BRAF* score, designed to identify *BRAF*^V600E^ PTCs (33), was the highest in the PTC cluster. A 12-gene signature designed to distinguish ATC tumor cells from PTC tumor cells (29) identified both ATC cells as well as stromal cells, in line with the dedifferentiated mesenchymal phenotype of ATC tumor cells (Supplemental Figure 1, D and E). Together, we generated an integrated thyroid cancer atlas that includes samples across a spectrum of thyroid cancer dedifferentiation.

### Identification of CAF and perivascular subpopulations.

**Figure 2:**
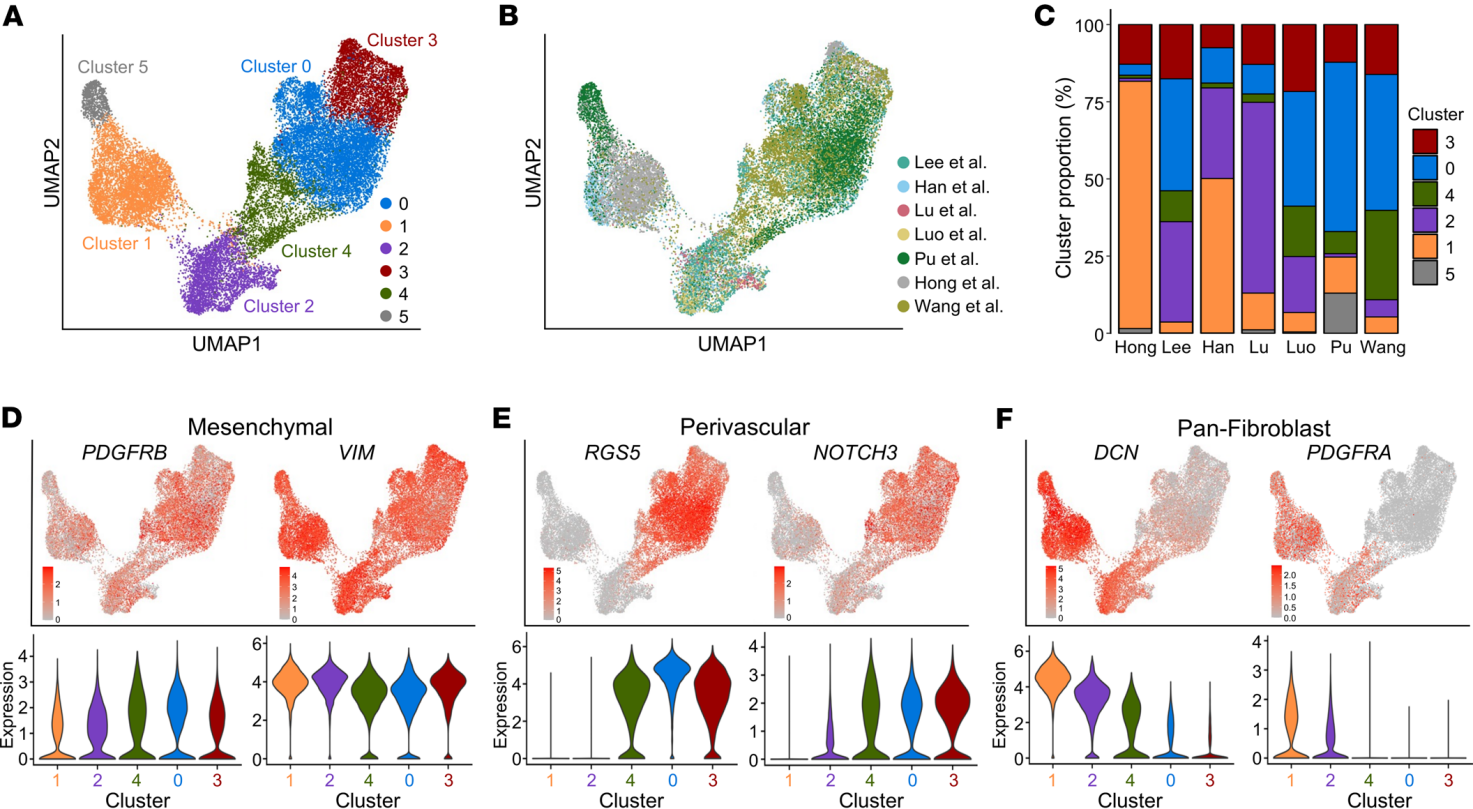
Stromal subclustering identifies broad CAF and perivascular populations. (**A** and **B**) UMAP showing subclustering of the broad stromal cluster from Figure 1B colored by (**A**) cluster and (**B**) paper. (**C**) Bar plots showing cluster proportion for each paper. (**D**–**F**) UMAP of stromal subclustering depicting expression of (**D**) mesenchymal marker genes, (**E**) perivascular marker genes, and (**F**) pan-fibroblast marker genes with violin plots below each UMAP showing expression by cluster. The width of each violin depicts the density of cells at each expression level.

**Figure 3:**
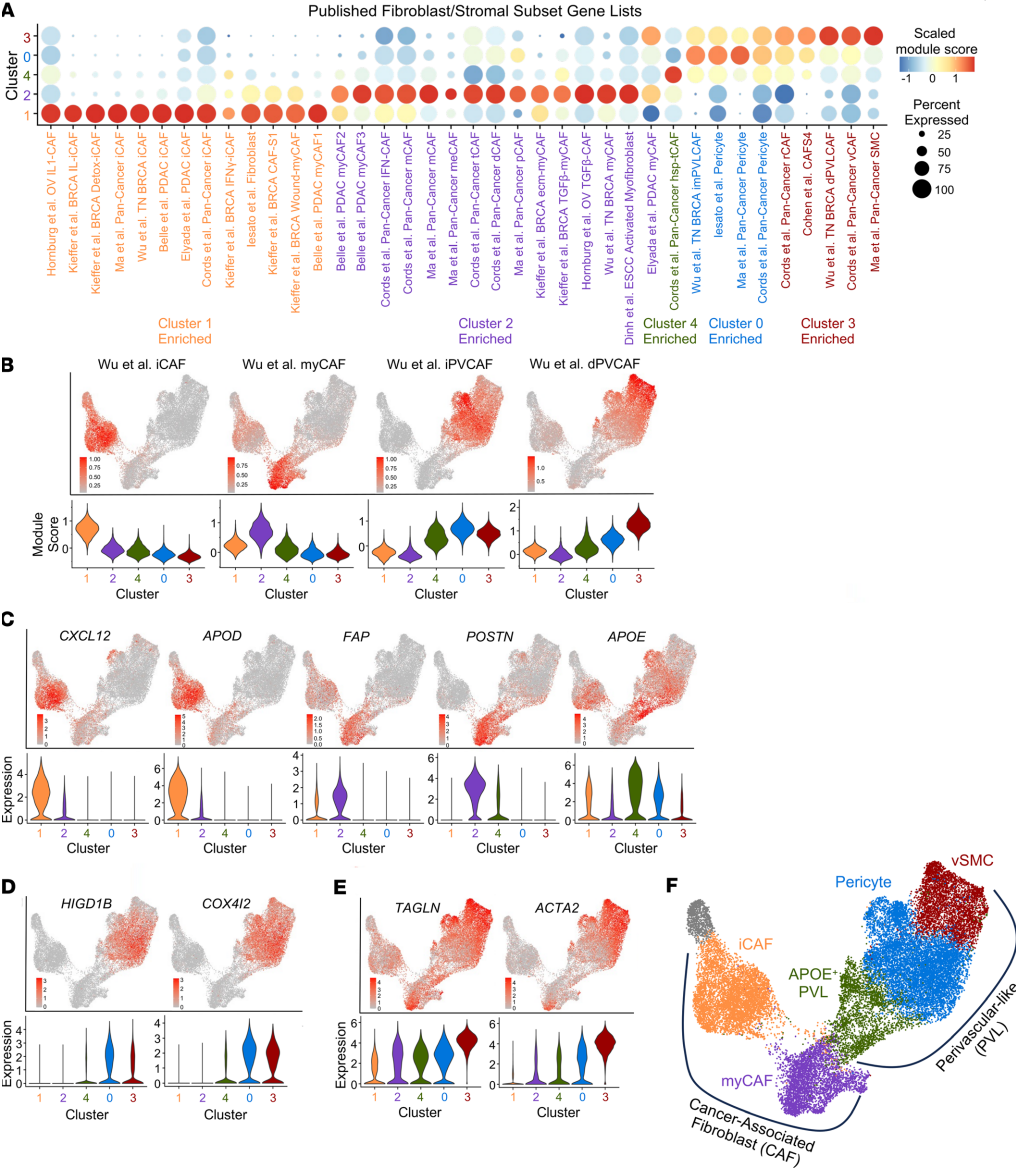
Defining CAF and perivascular subpopulations. (**A**) Scaled dot plot showing module scores for published CAF and perivascular populations by stromal subcluster. (**B**) UMAP of stromal subclustering showing module scores for CAF and perivascular-like (immature perivascular-like CAF, iPVCAF; differentiated perivascular-like CAF, dPVCAF) populations from Wu et al. (11) with violin plots below depicting expression by stromal subcluster. (**C**–**E**) UMAP showing marker genes of stromal subclusters with violin plots below. (**F**) Diagram outlining labeling of stromal subclustering into broad CAF and perivascular-like (PVL) groups with identification of individual clusters as iCAF, myCAF, APOE^+^ PVL, pericyte, and vSMC. OV, ovarian cancer; BRCA, breast cancer; TN BRCA, triple-negative breast cancer; PDAC, pancreatic ductal adenocarcinoma; ESCC, esophageal squamous cell carcinoma; mCAF, matrix CAF; meCAF, metabolic CAF; tCAF, tumor-like CAF; dCAF, dividing CAF; ecmCAF, extracellular matrix CAF; hsp-tCAF, heat shock protein tumor-like CAF.

To identify fibroblast subpopulations, we performed subclustering of 24,463 stromal cells in the single-cell atlas and identified 6 main clusters (Figure 2A). We proceeded with analysis of clusters 0–4, as cluster 5 was largely from 1 metastatic lymph node sample from Pu et al. (Figure 2, B and C, and Supplemental Figure 2A). We confirmed broad expression of *VIM* and *PDGFRB* across clusters, consistent with their mesenchymal phenotype (Figure 2D). We observed expression of perivascular markers *RGS5* and *NOTCH3* in clusters 4, 3, and 0 whereas pan-fibroblast markers *DCN* and *PDGFRA* were expressed mainly by clusters 1 and 2, with some expression in cluster 4 (Figure 2, E and F). We thus hypothesized that our stromal subclustering identifies both CAF and perivascular populations. To better classify these populations, we generated Seurat module scores (34) for published CAF and perivascular marker genes from diverse tumor types (8–15, 35, 36). Among hypothesized CAFs, cluster 1 showed enrichment of each iCAF gene set tested, whereas cluster 2 showed higher scores for matrix CAFs, myCAFs, and TGF-β–CAF gene sets. Within the hypothesized perivascular cell clusters, there was broad enrichment of perivascular gene sets, with cluster 3 having higher module scores for smooth muscle cells (SMCs) and cluster 0 having higher scores for pericyte gene sets. Cluster 4 showed enrichment for all perivascular gene sets but had no previously defined phenotype from the literature (Figure 3, A and B). We next performed differential gene expression analysis to identify marker genes of each stromal subcluster (Supplemental Table 2 and Supplemental Figure 2B). Top marker genes of clusters included known iCAF marker genes *CXCL12* and *APOD* (cluster 1), the myCAF gene periostin (*POSTN*) and the activated CAF gene fibroblast activation protein α (*FAP*) (cluster 2), *APOE* (cluster 4), pericyte markers *HIGD1B* and *COX4I2* (cluster 0), and SMC markers *ACTA2* and *TAGLN* (cluster 3). In addition to marking SMCs, *ACTA2* and *TAGLN* have been described as myCAF markers (13) and were also expressed by cluster 2 (Figure 3, C–E). Based on the concordance of published gene sets and our differential gene expression analysis, we labeled cluster 1 as iCAFs, cluster 2 as myCAFs, and cluster 0 as pericytes. Because cluster 3 expressed perivascular and SMC markers, we labeled it as vascular SMCs (vSMCs). We labeled cluster 4 as APOE^+^ perivascular-like (APOE^+^ PVL) based on its moderate expression of perivascular marker genes (Figure 3F). Gene ontology analysis of the marker genes for each population highlighted the broad roles of each population in matrix remodeling (Supplemental Figure 2C). Analysis of signaling pathway activation highlighted known fibroblast activating pathways TGF-β and WNT signaling in myCAFs, estrogen and androgen signaling in iCAFs, and tumor necrosis factor-related apoptosis-induced ligand (TRAIL) signaling in perivascular populations, consistent with the recently established role of TRAIL in facilitating crosstalk between endothelial cells and perivascular cells (Supplemental Figure 2D) (37). In conclusion, we identified myCAF, iCAF, and perivascular subpopulations that align with published gene sets for these populations.

### myCAFs are associated with aggressive tumors.

**Figure 4:**
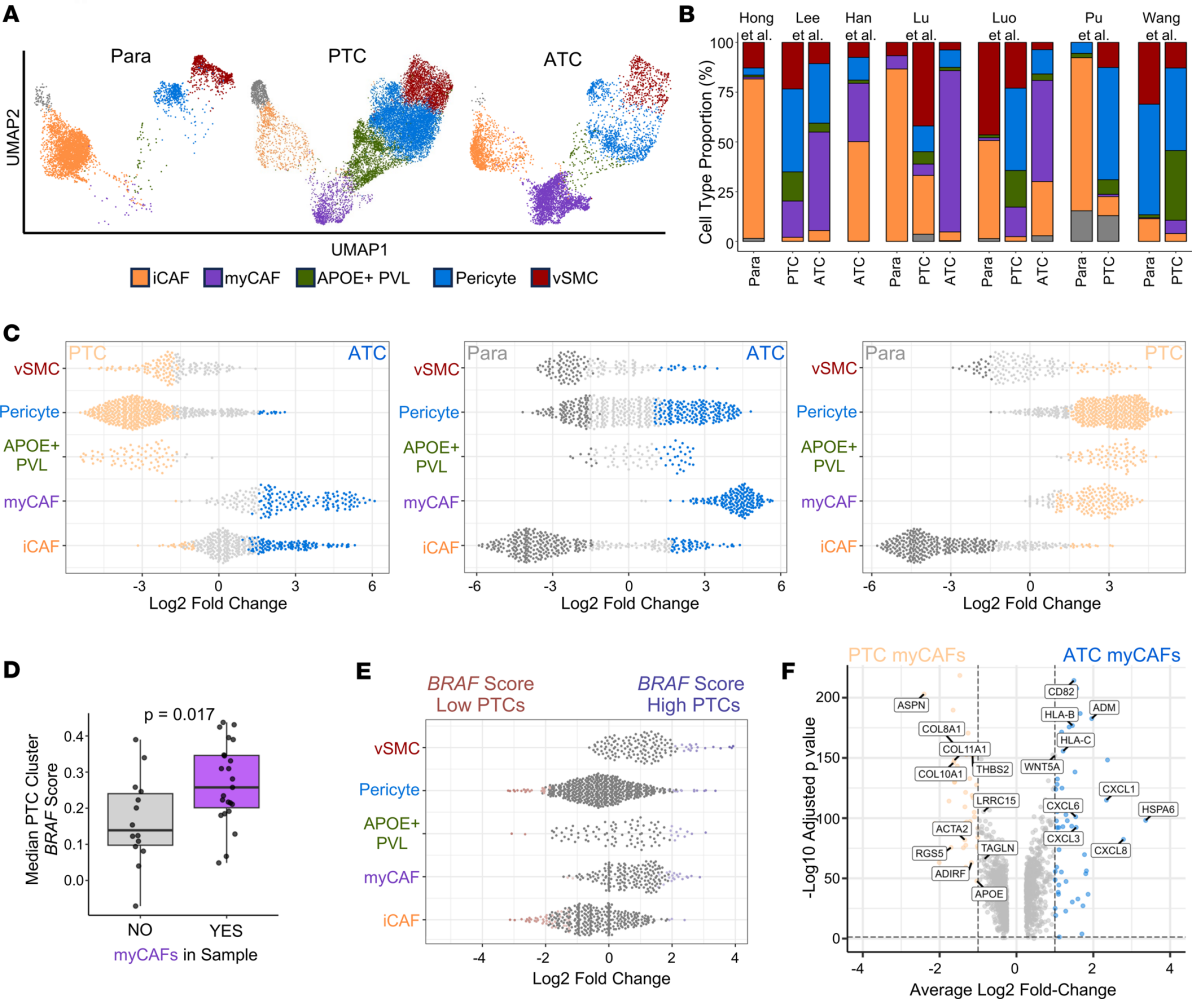
myCAFs are enriched in anaplastic and *BRAF*-like papillary thyroid cancer. (**A**) Stromal subclustering UMAP split by tumor histology (para, paratumor/normal samples). (**B**) Bar plots showing stromal cell type proportion for each tumor histology by paper. (**C**) Milo differential abundance testing of CAF and perivascular stromal cell populations between PTC and ATC samples (left), paratumor/normal and ATC samples (middle), and paratumor/normal and PTC samples (right). Individual dots depict neighborhoods calculated by Milo. Coloring of individual neighborhoods as dark gray (normal/paratumor), light orange (PTC), or blue (ATC) indicates differential abundance with a spatial false discovery rate (FDR) of less than 0.1. (**D**) Box plot depicting the median *BRAF* module score of PTC cells for individual PTC samples split by whether the sample contains myCAFs. *P* value calculated with Wilcoxon rank-sum test. (**E**) Milo differential abundance testing of CAF and perivascular stromal cell populations between PTC tumors split based on *BRAF* score high (median *BRAF* score for PTC cells within the tumor greater than 50th percentile) or *BRAF* score low (median *BRAF* score for PTC cells within the tumor less than 50th percentile). Coloring of individual neighborhoods as red (*BRAF* score low) or blue (*BRAF* score high) indicates differential abundance with a spatial FDR of less than 0.1. (**F**) Volcano plot showing differentially expressed genes between PTC myCAFs (left, light orange) and ATC myCAFs (right, blue). Colored dots indicate genes with an absolute log2 fold-change ≥ 1.0 and adjusted *P* < 0.05.

We next sought to determine the association of stromal subclusters with tumor histology and disease progression. We observed that myCAFs are predominantly found in malignant samples, with a subset of ATCs having the highest proportion of myCAFs (Figure 4, A and B, and Supplemental Figure 3A). Consistent with our prior work deconvoluting immune niches in bulk RNA-sequencing data (25), myCAF-rich tumors correlated with increased frequency of myeloid immune cells and anaplastic tumor cells (Supplemental Figure 3B). Differential abundance testing confirmed enrichment of myCAFs in malignant samples and ATCs and identified a robust pericyte signal in PTC. iCAFs showed enrichment in paratumors and in ATC relative to PTC (Figure 4C). iCAFs are commonly thought to be tumor distant (38), aligning with their presence in paratumor sampling. As previous studies have associated CAFs and desmoplastic stroma with *BRAF*^V600E^ PTCs (25, 39), we scored PTCs based on expression of genes from The Cancer Genome Atlas (TCGA) *BRAF*-*RAS* score (BRS) (33). PTCs with myCAFs had higher median *BRAF* scores than PTCs without myCAFs (*P* = 0.017), and PTCs with high median *BRAF* scores showed broad enrichment of stromal populations, including myCAFs (Figure 4, D and E). To determine how myCAF phenotypes shift as tumors dedifferentiate from PTC to ATC, we performed differential gene expression analysis of myCAFs based on sample histology. myCAFs in PTC showed a classic myofibroblast phenotype identified in pancreatic cancer (13, 38), with *ACTA2*, *TAGLN*, and collagen gene expression. *RGS5* was also upregulated in PTC myCAFs, suggesting a potential pericyte precursor for this population, consistent with the abundance of pericytes observed in differential abundance analysis and further supported by trajectory analysis (Figure 4F and Supplemental Figure 3, C and D). In contrast, ATC myCAFs exhibited upregulation of immune modulating genes (Figure 4F).

**Figure 5:**
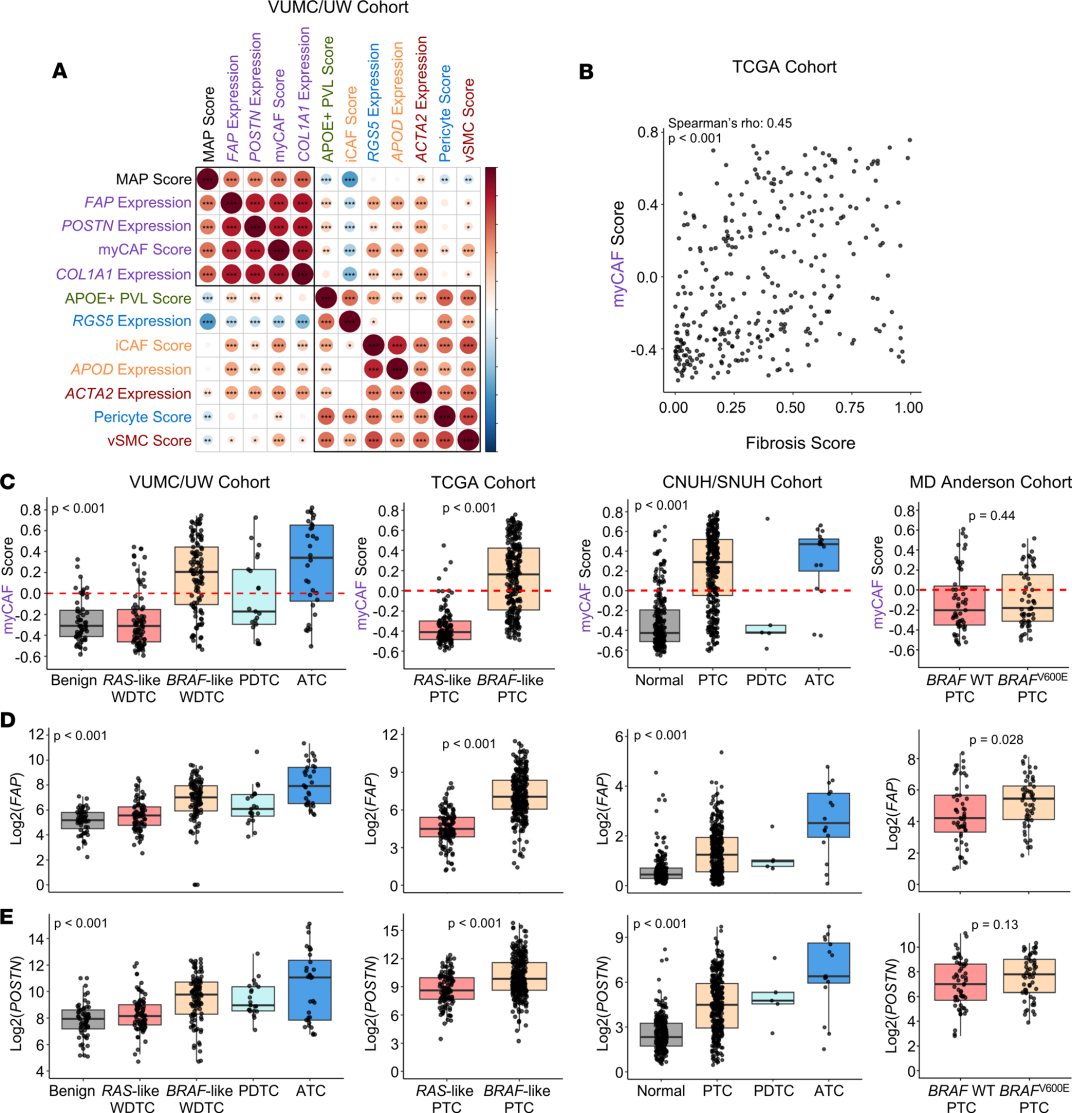
myCAF gene signatures are upregulated in malignant tumors across thyroid cancer bulk RNA-sequencing cohorts. (**A**) Corrplot showing Spearman’s rho correlations between single-sample GSVA (ssGSVA) scores for stromal subpopulations, ssGSVA MAP score, and expression of marker genes for stromal subpopulations in the Vanderbilt University/University of Washington bulk RNA-sequencing cohort (VUMC/UW cohort). Axes are ordered by hierarchical clustering. Boxes indicate hierarchical clustering groups. Significance levels indicate *P < 0.05, **P < 0.01, or ***P < 0.001. (**B**) Spearman’s rho correlation of myCAF ssGSVA score and a fibrosis score derived from H&E imaging data of TCGA PTCs (39). (**C**–**E**) Box plots showing (**C**) myCAF score or log2-transformed expression of CAF marker genes (**D**) *FAP* and (**E**) *POSTN* by diagnosis across 4 distinct bulk RNA-sequencing cohorts. *P* values calculated with Wilcoxon rank-sum test. Kruskal-Wallis test with subsequent pairwise Wilcoxon rank-sum tests with Bonferroni’s correction was used when comparing more than 2 groups. PDTC, poorly differentiated thyroid cancer; CNUH/SNUH, Chungnam/Seoul National University Hospitals.

**Figure 6:**
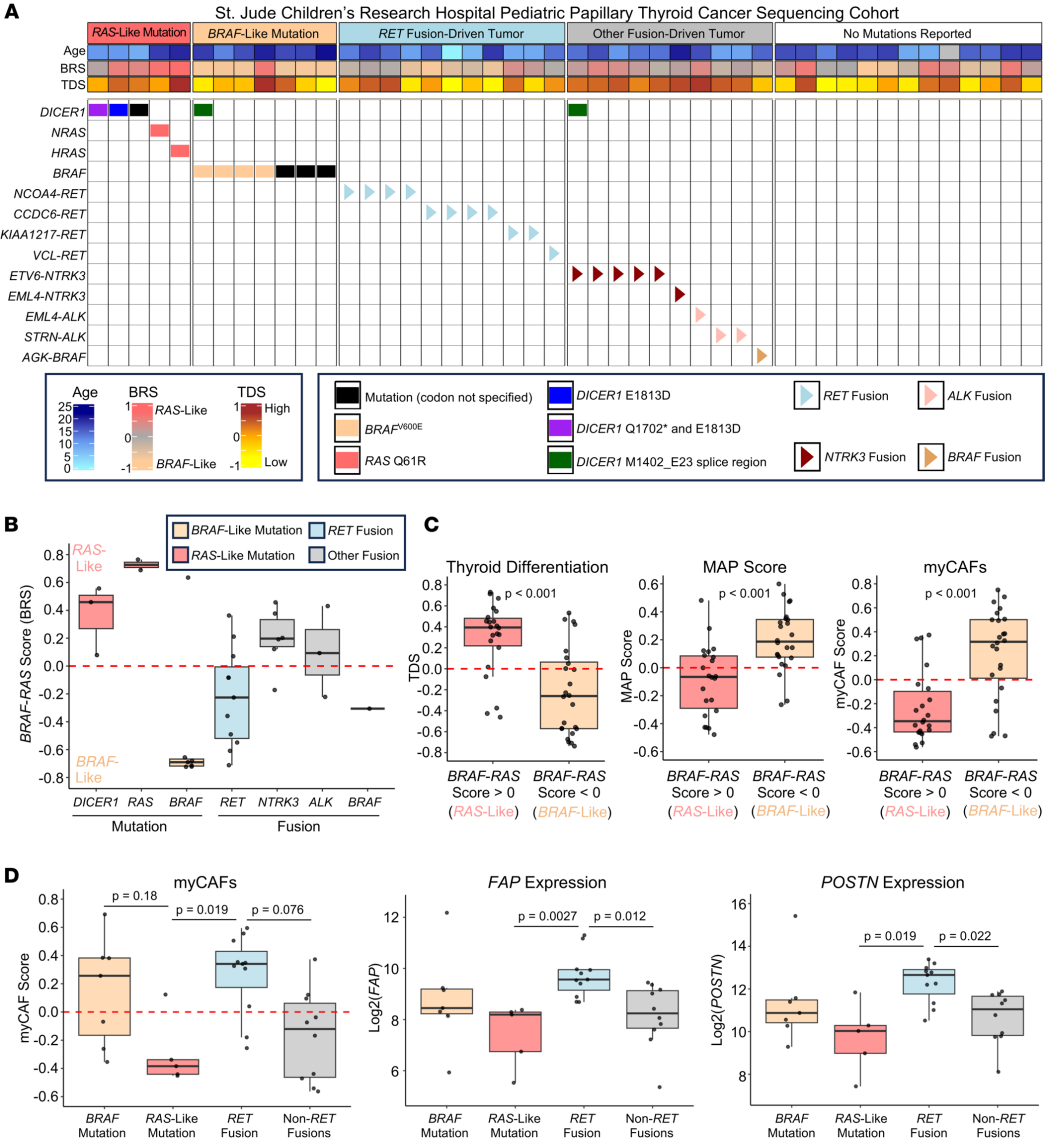
myCAFs are increased in pediatric thyroid cancers with *RET* fusions. (**A**) Oncoplot of St. Jude Children’s Research Hospital pediatric PTC sequencing cohort. (**B**) Box plots showing *BRAF*-*RAS* score (BRS) by driver mutation. (**C**) Box plots showing thyroid differentiation score (TDS, left), MAP score (middle), and myCAF score (right) by BRS categorization. *P* values calculated with Wilcoxon rank-sum test. (**D**) Box plots showing myCAF score (left), *FAP* expression (middle), and *POSTN* expression (right) by driver mutation category. *P* values calculated with Wilcoxon rank-sum test with Bonferroni correction.

To validate the association of myCAFs with aggressive tumors, we used single-sample gene set variation analysis (GSVA) to predict abundance of CAF and perivascular subpopulations in 4 large adult thyroid cancer bulk RNA-sequencing cohorts (25, 31, 33). We correlated stromal GSVA scores with our previously published MAP score, which identifies aggressive, fibroblast-rich tumors (25). Across cohorts, MAP score was correlated with myCAF GSVA scores and the expression of myCAF-associated genes (Figure 5A and Supplemental Figure 4A). To validate that myCAFs are enriched in tumors with increased stroma and matrix remodeling, we identified a positive correlation (Spearman’s rho 0.45, *P* < 0.001) between myCAF score in the TCGA cohort and a fibrosis score previously generated from histology imaging data (Figure 5B) (39). Next, we assessed the predicted abundance of stromal cells across benign, WDTC, and ATC in these cohorts. myCAF scores and myCAF marker genes were upregulated in *BRAF*-like WDTCs and ATCs across VUMC/UW, TCGA, and Chungnam/Seoul National University Hospitals (CNUH/SNUH) cohorts. Aggressive PTCs from MD Anderson showed increased *FAP* expression in *BRAF*^V600E^ tumors (*P* = 0.028) (Figure 5, C–E). Additionally, we tested myCAF abundance in a pediatric thyroid cancer sequencing cohort from St. Jude’s Research Hospital (Figure 6A). In contrast with adult thyroid cancers, pediatric thyroid cancers are commonly driven by fusions, with fusion-driven disease having an increased risk of metastasis and treatment resistance relative to *BRAF*- or *RAS*-mutated tumors (40, 41). Despite these genetic differences, characterizing pediatric thyroid cancers by BRS showed an association between *BRAF*-like pediatric thyroid cancers and myCAFs. *BRAF*-like pediatric tumors were also associated with higher MAP scores and lower TDS, consistent with previous studies in adult cohorts (Figure 6, B and C) (25, 33, 42). By mutation, *RET* fusion-driven tumors had increased myCAFs and expression of *POSTN* and *FAP* relative to non-*RET* fusion-driven tumors (Figure 6D). Together, we identified enrichment of myCAFs in *BRAF*-like tumors across both adult and pediatric thyroid cancers.

In contrast with myCAFs, iCAFs and perivascular subpopulations did not show clear shifts in predicted abundance with tumor dedifferentiation (Supplemental Figure 4, B–E). iCAFs were increased in VUMC/UW ATCs relative to WDTCs (*P* = 0.0019) and in *BRAF*-like WDTCs in TCGA cohort relative to *RAS*-like WDTCs (*P* < 0.001), but the CNUH/SNUH cohort showed the highest predicted iCAF levels in benign thyroids. However, many benign and malignant surgically removed thyroids have background autoimmune inflammatory processes (e.g., Hashimoto thyroiditis). To assess whether increased iCAF infiltrate in benign thyroids was related to autoimmune disease, we evaluated the predicted abundance of iCAFs in benign lesions from the VUMC/UW cohort that underwent detailed pathologic and clinical evaluation. Among VUMC/UW benign thyroids, there was a higher predicted abundance of iCAFs (*P* = 0.0015), but not myCAFs, in patients with confirmed Hashimoto thyroiditis, as compared with non-autoimmune benign thyroid nodules (Supplemental Figure 4F). These data indicate that autoimmune conditions may have fibroblasts with inflammatory phenotypes similar to iCAFs.

**Figure 7:**
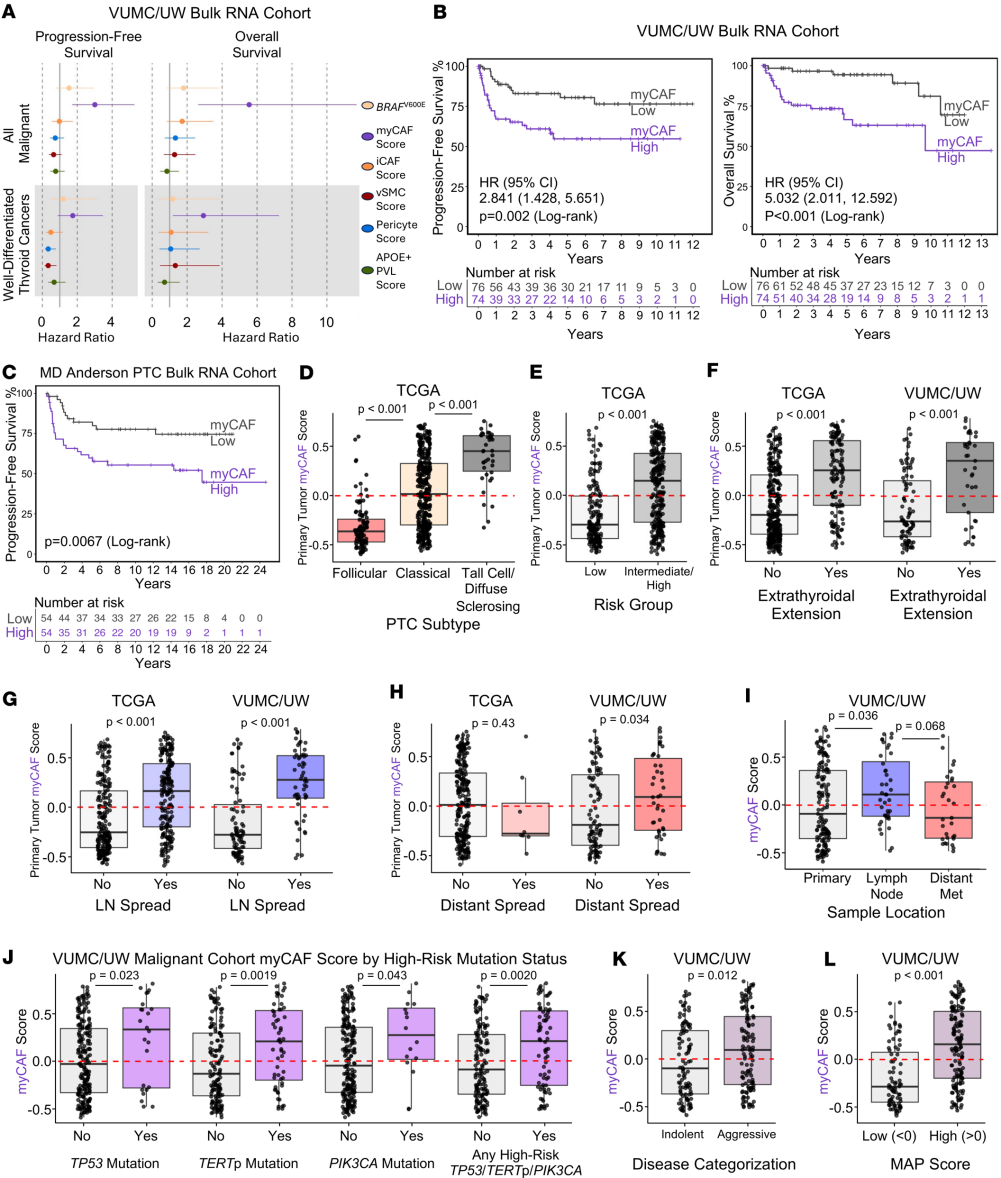
myCAFs are associated with aggressive thyroid cancer tumor phenotypes. (**A**) PFS (left) and OS (right) forest plots with IQR hazard ratios and 95% confidence intervals for the VUMC/UW bulk RNA-sequencing cohort split into malignant samples (top) or WDTCs (bottom). Hazard ratios are shown for *BRAF*^V600E^ mutation and stromal cell ssGSVA scores. (**B**) PFS (left) and OS (right) survival curves showing the VUMC/UW malignant cohort split into myCAF-high/low by 50th percentile myCAF score. (**C**) PFS curve of MD Anderson PTC bulk RNA-sequencing cohort split into myCAF-high/low by 50th percentile myCAF score. *P* values for **B** and **C** calculated with log-rank test. (**D**–**H**) Box plots depicting primary tumor myCAF score in (**D**) TCGA PTCs stratified by histology subtype, (**E**) TCGA PTCs stratified by risk group as defined by the 2009 American Thyroid Association guidelines (43), (**F**) TCGA PTCs (left) and VUMC/UW malignant cohort (right) stratified by the presence or absence of extrathyroidal extension, (**G**) TCGA PTCs (left) and VUMC/UW malignant cohort (right) stratified by the presence or absence of lymph node spread, and (**H**) TCGA PTCs (left) and VUMC/UW malignant cohort (right) stratified by the presence or absence of distant metastasis. (**I**–**L**) myCAF score in the VUMC/UW malignant cohort stratified by (**I**) sample location, (**J**) presence or absence of indicated high-risk mutations, (**K**) disease categorization into indolent or aggressive as previously defined (25), and (**L**) MAP score. *P* values for **D**–**L** calculated with Wilcoxon rank-sum test with Bonferroni correction when more than 2 groups tested.

We next assessed the association of stromal populations with features of aggressive disease and patient outcomes. In malignant samples in the VUMC/UW cohort, myCAF score was associated with worse progression-free survival (PFS) and overall survival (OS; Figure 7, A and B). We also observed worse PFS in myCAF-high tumors in a cohort of aggressive PTCs (*P* = 0.045; Figure 7C), indicating that decreases in survival in myCAF-rich tumors are not solely driven by ATC. TCGA cohort was designed to consist of indolent PTCs with few survival events (33). However, we identified that TCGA PTCs with aggressive histologic subtypes (tall cell or diffuse sclerosing) had the highest myCAF scores (*P* < 0.001, Figure 7D), and “intermediate- or high-risk” PTCs, as designated by the 2009 American Thyroid Association guidelines (43), had higher myCAF scores than “low-risk” PTCs (*P* < 0.001, Figure 7E). Additionally, in both TCGA and VUMC/UW cohorts, tumors with extrathyroidal extension (*P* < 0.001 TCGA; *P* < 0.001 VUMC/UW) or lymph node metastases (*P* < 0.001 TCGA; *P* < 0.001 VUMC/UW) had higher myCAF scores (Figure 7, F and G). Increased myCAF score in primary tumors with distant metastases was also observed in the VUMC/UW cohort (*P* = 0.034; Figure 7H), and lymph node metastases had higher myCAF scores than primary malignant tumors in the VUMC/UW cohort (*P* = 0.036; Figure 7I). Finally, we identified increased myCAFs in tumors with known high-risk mutations as well as in tumors with aggressive disease (*P* = 0.012) and high MAP scores (*P* < 0.001), as categorized by our previous work (Figure 7, J–L) (25). In total, we identified myCAFs as enriched in malignant samples and ATCs across our single-cell atlas and identified association with ATC, PFS, OS, and aggressive WDTC features in bulk RNA-sequencing cohorts.

### myCAFs are enriched in epithelial ATCs but not mesenchymal ATCs.

**Figure 8:**
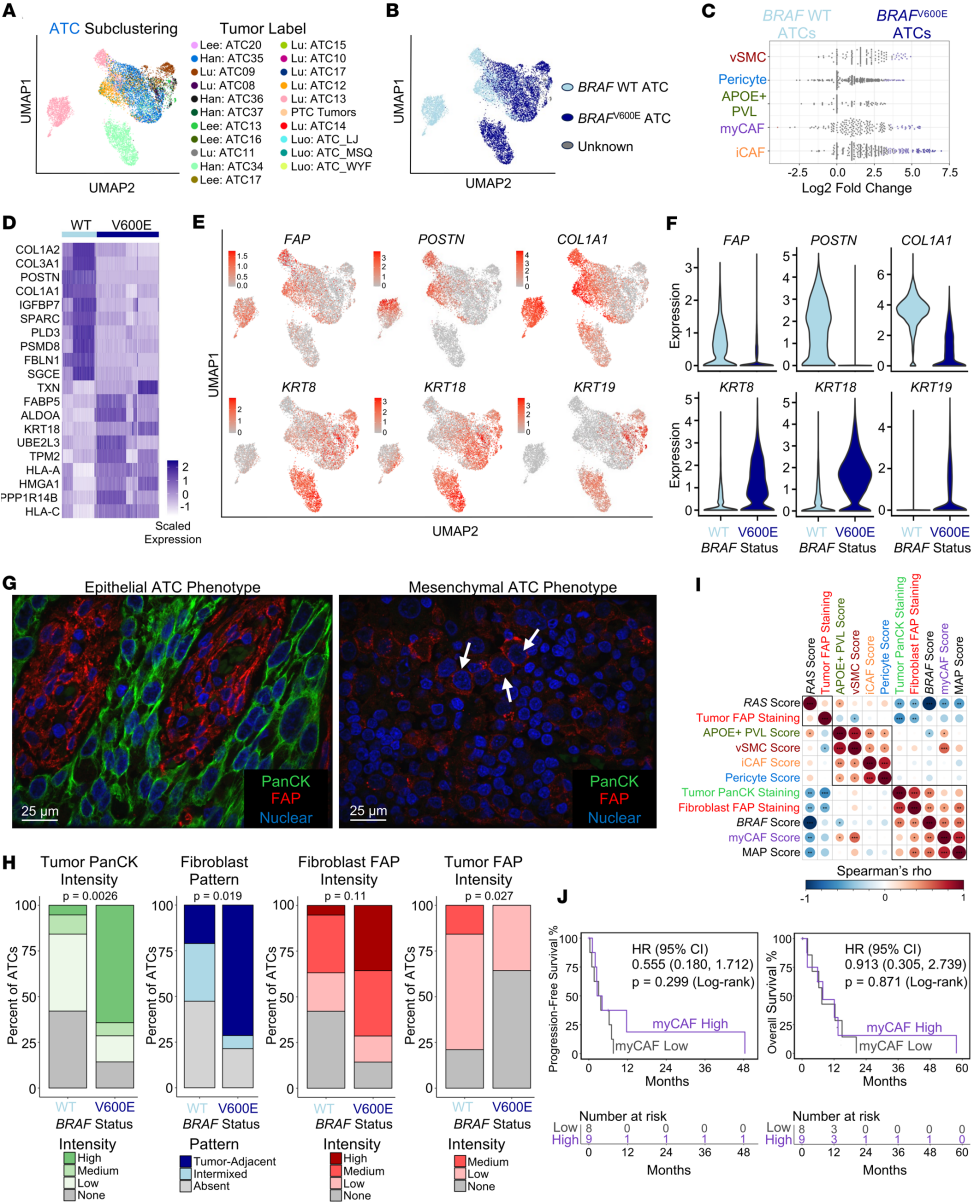
myCAFs are enriched in epithelial ATCs. (**A** and **B**) UMAP showing subclustering of ATC tumor cells colored by (**A**) sample (tumor label) or (**B**) *BRAF*^V600E^ mutation status. (**C**) Milo differential abundance testing of CAF and perivascular stromal cell populations between *BRAF* WT and *BRAF*^V600E^ ATC tumors. Individual dots depict neighborhoods calculated by Milo. Coloring of individual neighborhoods as dark blue (*BRAF*^V600E^) or light blue (*BRAF* WT) indicates differential abundance with a spatial FDR of less than 0.1. (**D**) Heatmap showing scaled expression of the top 10 differentially expressed genes with expression in at least 80% of cells in the population of interest between *BRAF* WT and *BRAF*^V600E^ ATCs. (**E**) UMAP of ATC subclustering colored by expression of myCAF genes (top row) or epithelial keratins (bottom row). (**F**) Violin plots of expression of genes from **E** in ATC tumor cells split by *BRAF* status. (**G**) Representative multiplex immunofluorescence images from staining of 33 ATCs in the VUMC/UW cohort for pan-cytokeratin (PanCK, green) and FAP (red). Left: representative image of ATC with PanCK^+^ tumor cells and tumor-adjacent FAP^+^ fibroblasts. Right: representative image of ATC with no PanCK staining, FAP^+^ tumor cells, and minimal stromal FAP staining. White arrows point to malignant nuclei with membranous FAP staining. (**H**) Bar plots showing pathologist scoring of multiplex immunofluorescence. Tumors are split by *BRAF* status. *P* values calculated with Fisher’s exact test. (**I**) Corrplot showing Spearman’s rho correlations for ATC multiplex immunofluorescence staining samples comparing PanCK tumor cell intensity, FAP tumor cell intensity, FAP fibroblast intensity, ssGSVA scores for stromal subpopulations, ssGSVA MAP score, *BRAF* score, and *RAS* score in the VUMC/UW ATC cohort. Axes ordered by hierarchical clustering. Boxes indicate hierarchical clustering groups. Significance levels indicate *P < 0.05, **P < 0.01, or ***P < 0.001. (**J**) PFS (left) and OS (right) survival curves showing VUMC/UW ATCs split by 50th percentile myCAF score. *P* values calculated by log-rank test.

Our previous work utilizing MAP score initially identified 2 ATC subsets with unique stromal infiltration, high-MAP ATCs with robust CAF infiltrate and low-MAP ATCs with minimal CAF infiltrate (25). To further refine these subsets and investigate which ATCs are associated with myCAF infiltrate, we performed subclustering of the ATC tumor cell clusters in our single-cell atlas. ATC tumor cells largely clustered by sample, consistent with the heterogeneity in gene expression and copy number alterations that is a hallmark of ATC (Figure 8A) (20, 28). However, distinguishing clusters by the presence or absence of *BRAF*^V600E^ mutation indicated that despite the heterogeneity of these tumors, *BRAF*^V600E^ and *BRAF* WT ATCs have distinct gene expression patterns (Figure 8B). Through differential abundance and differential gene expression analysis, we identified epithelial ATCs, enriched for high-MAP tumors with *BRAF*^V600E^, and mesenchymal ATCs, enriched for low-MAP tumors and *BRAF* WT tumors. Specifically, we identified that epithelial ATCs are often *BRAF*^V600E^ mutant with a more epithelial, keratin-rich gene expression program and a robust CAF infiltrate. Rather than having robust CAF recruitment, mesenchymal ATCs are typically *BRAF* WT ATCs that demonstrate a highly mesenchymal gene program, expressing their own array of extracellular matrix genes (Figure 8, C–F, and Supplemental Figure 5, A–C). As identified by Lu et al., mesenchymal ATCs also express *CDK6* (28). Consistent with their mesenchymal gene expression, TGF-β signaling was activated in *BRAF* WT ATCs whereas MAPK signaling was activated in *BRAF*^V600E^ ATCs (Supplemental Figure 5D). To confirm that these *BRAF* WT mesenchymal tumor cells are not fibroblasts, we performed copy number inference (44) and observed *FAP*-expressing clusters with aneuploid tumor cells. In contrast, *BRAF*^V600E^ tumors had diploid clusters of *FAP*-expressing CAFs (Supplemental Figure 5, E–H).

To morphologically visualize and confirm these differences, we performed multiplex immunofluorescence for pan-cytokeratin and FAP across 33 ATCs (19 *BRAF* WT, 14 *BRAF*^V600E^) from the VUMC/UW bulk RNA-sequencing cohort. We histologically confirmed these 2 distinct ATC patterns: (a) epithelial ATCs had pan-cytokeratin-expressing tumor cells with extensive FAP^+^ tumor-adjacent stroma (epithelial ATCs); and (b) mesenchymal ATCs had pan-cytokeratin-negative tumor cells with frequent FAP expression and few fibroblasts (mesenchymal ATCs) (Figure 8G). Quantification indicated that *BRAF*^V600E^ tumors had greater tumor pan-cytokeratin intensity (*P* = 0.0026), a tumor-adjacent fibroblast pattern (*P* = 0.019), and a trend toward greater fibroblast FAP intensity (*P* = 0.11). In contrast, *BRAF* WT tumors had greater intensity of FAP tumor staining (*P* = 0.027), indicating that *BRAF*^V600E^ ATCs were predominantly “epithelial” whereas *BRAF* WT were “mesenchymal,” though the relationship was not mutually exclusive (Figure 8H).

We next correlated these staining data to MAP score and stromal GSVA scores. MAP score previously separated ATCs into CAF-rich (MAP-high) and CAF-depleted (MAP-low) tumors (25). We observed that ATC fibroblast FAP staining had a significant correlation with myCAF score, MAP score, and greater *BRAF*-like phenotype, but not other stromal populations. Pan-cytokeratin intensity had a significant correlation with *BRAF*-like phenotype (Figure 8I). Although our survival analysis across bulk RNA-sequencing cohorts indicates that CAF-rich tumors have worse outcomes, that may not be the case within universally aggressive ATCs (19). When comparing PFS and OS between myCAF-high and myCAF-low ATCs, we observed no difference, and multivariable Cox regression indicated differential interaction between myCAFs and outcome by tumor histology (Figure 8J and Supplemental Figure 6). Importantly, this is a historic, retrospective ATC cohort, and these patients did not receive targeted therapy or immunotherapy. Overall, we have identified 2 distinct ATCs: an epithelial ATC, commonly *BRAF*^V600E^, with keratin expression and fibroblast infiltrate, and a mesenchymal ATC with few fibroblasts.

### Spatial mapping of CAFs in tumor progression.

Based on the clinical relevance of CAFs in tumor progression, we investigated the spatial localization of CAF populations. We generated spatial transcriptomic data across the spectrum of disease progression, including 8 *RET* fusion-driven pediatric PTCs, 5 *BRAF*^V600E^ PTCs, and 15 ATC samples, 7 of which spatially capture WDTC as it dedifferentiates into ATC (Supplemental Figure 7, A and B). In *BRAF* WT ATCs, we observed ubiquitous *CDK6* expression and heterogeneous expression of CAF markers in tumor cell regions, consistent with tumor cell expression of a CAF-like mesenchymal profile (Supplemental Figure 7, C–E).

**Figure 9:**
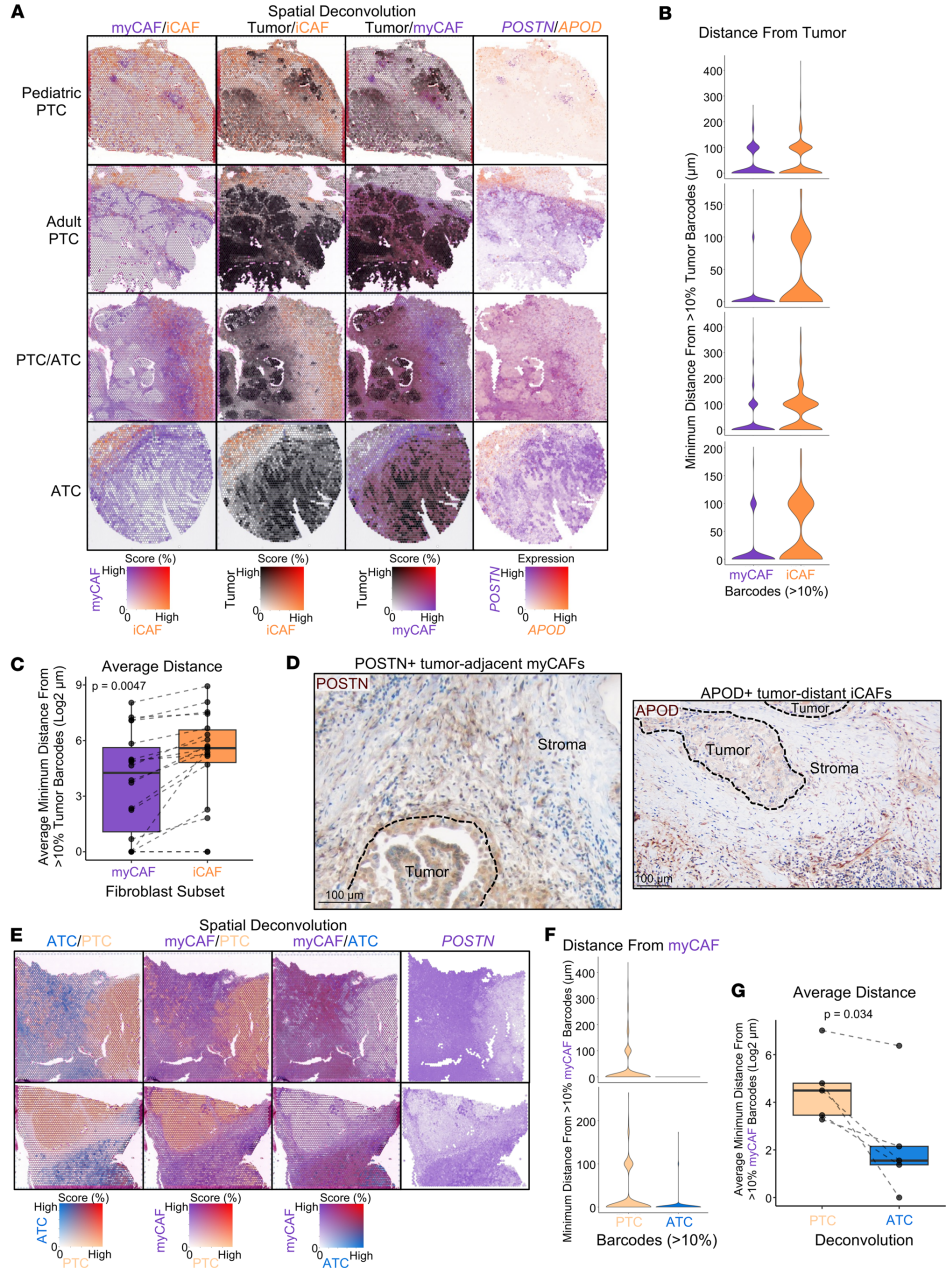
Spatial localization of CAF populations in thyroid cancer progression. (**A**) Spatial feature plots of myCAF (purple), iCAF (orange), and tumor cell (black) RCTD scores and myCAF (*POSTN*, purple) and iCAF (*APOD*, orange) marker gene expression. Tumor cell deconvolution is the sum of PTC and ATC deconvolution. From top to bottom samples shown are a pediatric PTC (Peds04), adult PTC (Thy7), adult PTC/ATC (Thy5), and adult ATC (Thy4). (**B**) Violin plots pertaining to samples from **A** showing the minimum Euclidean distance from a spatial barcode with at least 10% tumor RCTD for each spatial barcode with at least 10% myCAF RCTD (purple) or iCAF RCTD (orange). (**C**) Box plot showing the average of spatial barcode minimal Euclidean distances from **B** for myCAF (purple) and iCAF (orange) across 18 spatial transcriptomics samples with *BRAF*^V600E^ or a *RET* fusion and the presence of both myCAF and iCAF RCTD populations. *P* value calculated with paired *t* test. (**D**) Representative images of IHC for POSTN (left) and APOD (right) at the invasive edge of PTC. (**E**) Spatial feature plots of 2 mixed PTC/ATC samples (Thy6, Thy11) showing ATC (blue), PTC (light orange), and myCAF (purple) RCTD or myCAF (*POSTN*, purple) marker gene expression. (**F**) Violin plots pertaining to samples from **E** showing the minimum Euclidean distance from a spatial barcode with at least 10% myCAF RCTD for each spatial barcode with at least 10% PTC RCTD (light orange) or ATC RCTD (blue). (**G**) Box plot showing the average of spatial barcode minimal Euclidean distances from **F** for PTC (light orange) and ATC (blue) across 5 mixed PTC/ATC tumors with *BRAF*^V600E^ mutations. *P* value calculated with paired *t* test.

Because our spatial transcriptomics data contain multiple cells for each spatial barcode, we used our single-cell atlas to dissect the localization of individual cell types as a percentage of each spatial barcode. Spatial mapping of tumor cell and CAF subpopulations revealed a striking pattern of tumor-adjacent myCAFs and tumor-distant iCAFs across pediatric PTCs, adult PTCs, and ATCs (Figure 9A). We quantified the minimum distance of myCAFs and iCAFs from tumor cells in PTCs and *BRAF*^V600E^ ATC samples containing both myCAFs and iCAFs (*n* = 18). iCAFs were farther from tumor cells across samples as compared with myCAFs (*P* = 0.0047, Figure 9, B and C). IHC for the myCAF protein POSTN and the iCAF protein APOD confirmed the tumor-adjacent myCAF and tumor-distant iCAF localization and the increased abundance of both populations in PTC and ATC stroma relative to stroma in benign thyroid nodules (Figure 9D and Supplemental Figure 8, A–D). Additionally, APOD staining was observed in the stroma of Hashimoto thyroiditis (Supplemental Figure 8E). In 1 pediatric PTC with background Hashimoto thyroiditis, we observed spatial mapping of iCAFs to surrounding areas of lymphocytic thyroiditis, and 2 single-cell paratumor samples with background Hashimoto thyroiditis had interactions between iCAFs and immune cell populations, further supporting fibroblast infiltrates with iCAF-like inflammatory phenotypes in thyroid autoimmune disease (Supplemental Figure 9).

**Figure 10:**
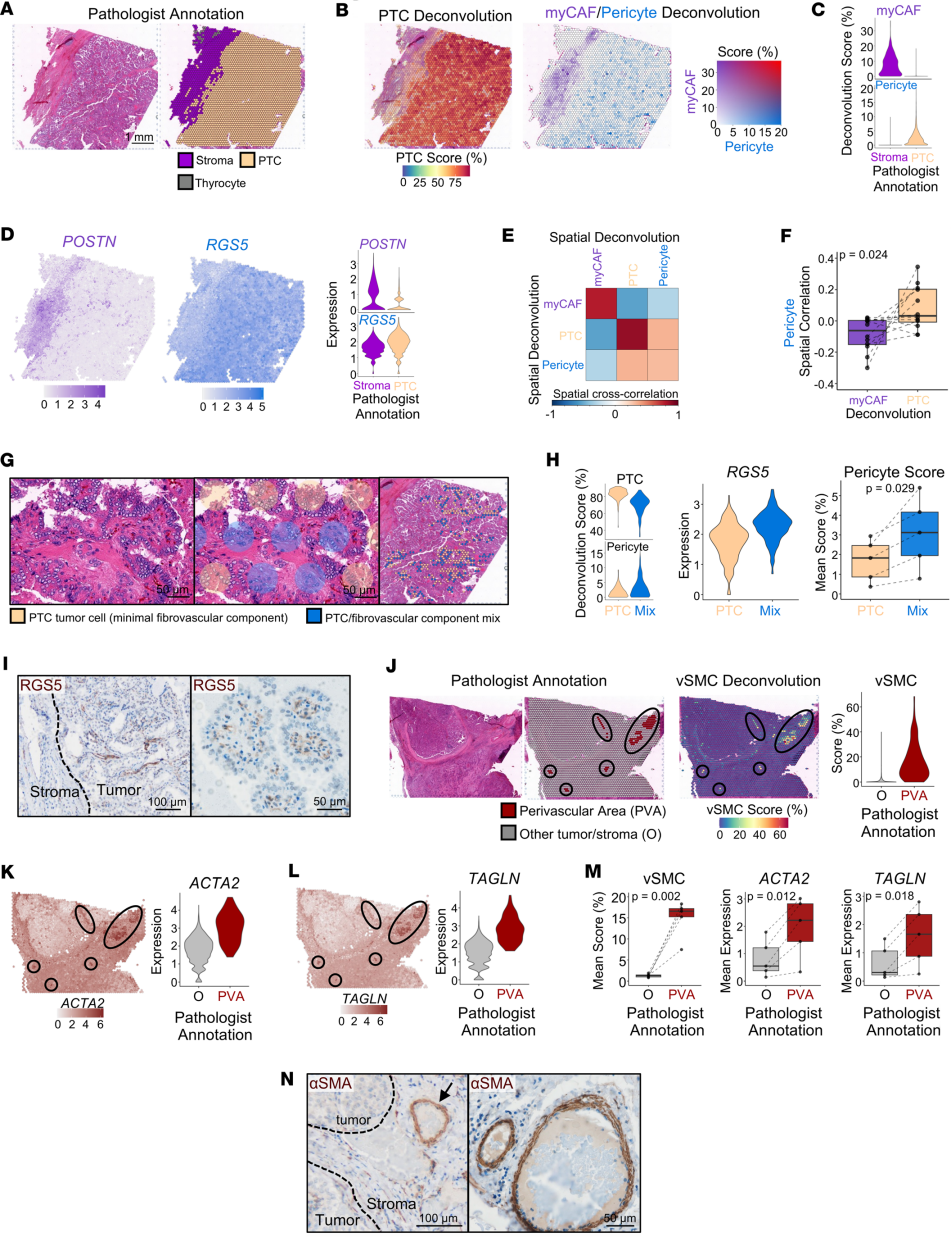
Spatial localization of perivascular populations in thyroid cancer. (**A**–**E**) Spatial analysis of representative PTC Thy15 showing (**A**) pathologist annotation of PTC and stromal spatial barcodes, (**B**) spatial feature plots of PTC and myCAF/pericyte RCTD scores, (**C**) violin plots of myCAF and pericyte RCTD scores stratified by pathologist annotation, (**D**) spatial feature plots and violin plots depicting myCAF (POSTN) and pericyte (RGS5) marker gene expression stratified by pathologist annotation, and (**E**) a heatmap of MERINGUE spatial cross-correlation of myCAF, PTC, and pericyte RCTD scores (ordered by hierarchical clustering). (**F**) Box plot showing pericyte spatial cross-correlation with myCAF and PTC across 12 PTC samples containing PTC and stromal regions. *P* value calculated with paired *t* test. (**G**) Pathologist annotation of representative PTC regions into predominantly tumor cell or a mix of tumor and fibrovascular cells. Left/middle: zoomed-in hematoxylin and eosin staining (left) with labeled spatial barcodes (middle). Right: zoomed-out hematoxylin and eosin staining with a sample of the spatial barcodes labeled. (**H**) Violin plots showing PTC and pericyte RCTD scores (left) and pericyte marker RGS5 expression (middle) stratified by pathologist annotation from **G**. Right: box plots depicting average pericyte RCTD deconvolution scores in PTC or PTC/fibrovascular component mix regions across five samples (Thy15-17, Peds07-08). *P* value calculated with paired *t* test. (**I**) Representative RGS5 IHC of PTC. (**J**) Pathologist spatial annotation of large perivascular areas (PVAs) (left) and spatial feature plot of vSMC RCTD scores (middle). Right: violin plot of vSMC RCTD score stratified by pathologist large PVA annotation. (**K** and **L**) Spatial feature plots (left) and associated violin plots (right) for vSMC marker genes (**K**) *ACTA2* and (**L**) *TAGLN*. (**M**) Box plots showing average vSMC RCTD score (left) or vSMC marker gene expression (right) for 5 tumors stratified by pathologist annotation (Supplemental Figure 10F). *P* values calculated with paired *t* test. (**N**) IHC of αSMA in a representative PTC. Black arrow highlights a large PVA.

To comprehensively understand the stromal picture of thyroid tumors, we explored the spatial localization of myCAFs, pericytes, and vSMC. For evaluation of myCAFs, we assessed 5 *BRAF*^V600E^ samples with PTC tumor cells dedifferentiating into ATC. We observed abundant myCAFs with *POSTN* expression infiltrating ATC regions but only lining the periphery of PTC regions (Figure 9E and Supplemental Figure 10A). Distance quantification indicated closer proximity of ATC cells to myCAFs across all 5 tumors (*P* = 0.034; Figure 9, F and G), quantitatively confirming increased myCAF infiltration as tumors progress from PTC to ATC. Because pericytes were upregulated in PTC single-cell samples, we next evaluated PTC specimens with adjacent stroma. As expected, myCAFs mapped to the invasive perimeter of PTC areas. In contrast with myCAFs, pericytes mapped within central areas of PTC (Figure 10, A–D, and Supplemental Figure 10, B–E). This association with pericytes was quantitatively confirmed using spatial cross-correlation analysis across 12 PTCs with mapping of both pericyte and myCAF populations (*P* = 0.024; Figure 10, E and F). Pathologist annotation of PTC regions demonstrated pericyte deconvolution was concentrated within the papillary vascular cores of PTC (*P* = 0.029; Figure 10, G and H). IHC further confirmed RGS5^+^ pericytes within the papillary fibrovascular core of PTCs (Figure 10I). vSMCs localized to large perivascular areas (pathologist annotated) on 5 tumors (Figure 10, J–M, and Supplemental Figure 10, F–H). IHC confirmed αSMA^+^ vSMCs lining large vessels within tumors (Figure 10N). In conclusion, we spatially mapped key stromal cell populations in thyroid cancer and identified a robust increase in tumor-adjacent myCAFs with dedifferentiation in *BRAF*^V600E^ tumors.

### myCAFs abut invasive tumor cells.

**Figure 11:**
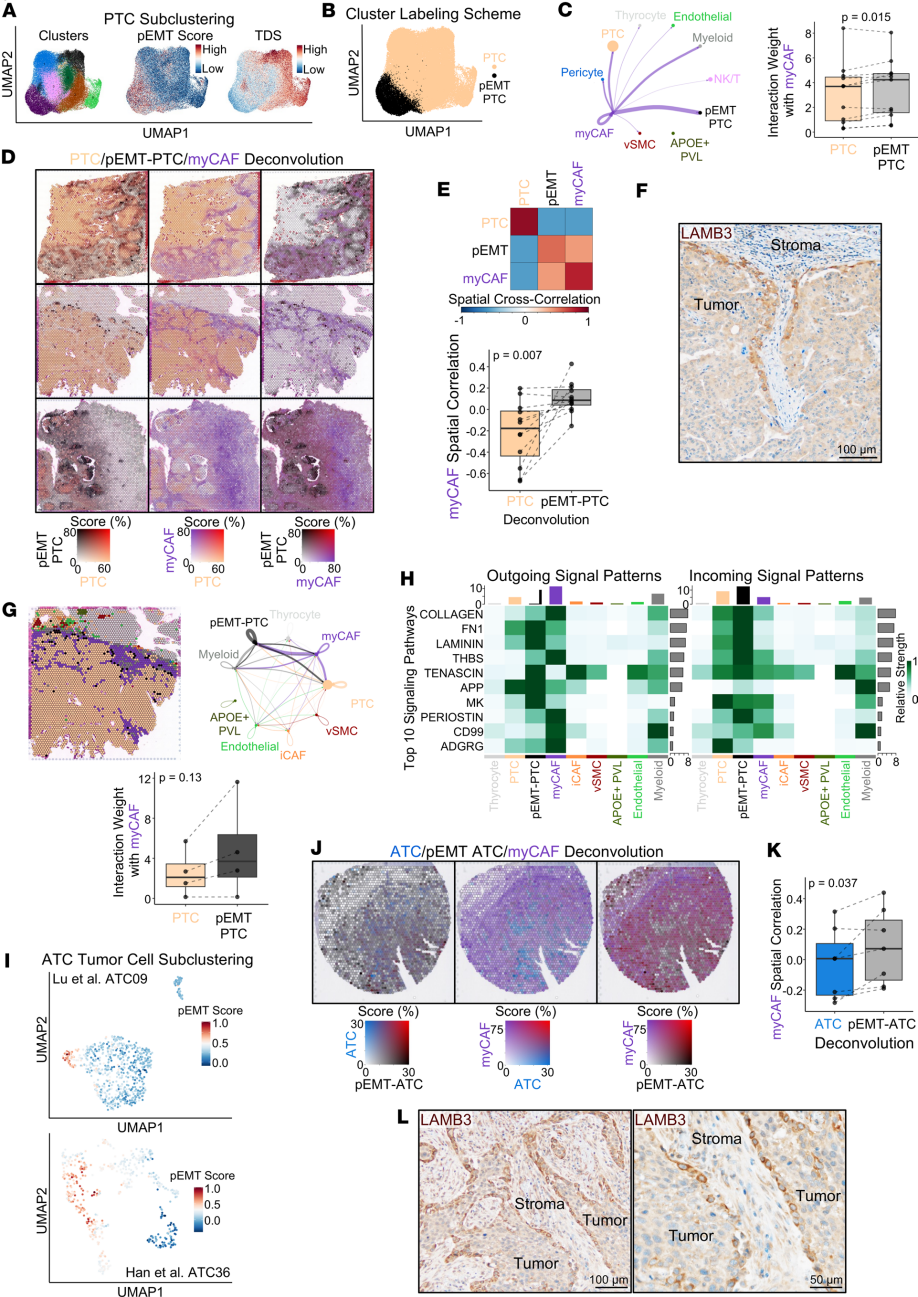
Spatial mapping reveals a pEMT phenotype in invasive tumor cells. (**A**) UMAP of PTC tumor cell subclustering showing subclusters, pEMT score, and TDS. (**B**) UMAP labeling PTC subclusters as PTC or pEMT-PTC. (**C**) Interaction weights between myCAFs and other populations in PTC samples. Left: representative PTC WJL from Luo et al. Line widths depict interaction weights. Right: Box plot depicting interaction weights between myCAFs and PTC or pEMT-PTC in 11 PTC samples containing each population. *P* value calculated with paired *t* test. (**D**) Spatial feature plots of PTC, pEMT-PTC, and myCAF RCTD scores for representative pediatric PTC (Peds08, top), adult PTC (Thy7, middle), and adult mixed PTC/ATC (Thy5, bottom). (**E**) Spatial cross-correlation analysis between myCAF, PTC, and pEMT-PTC RCTD scores. Top: heatmap showing spatial cross-correlation ordered by hierarchical clustering for representative PTC Peds08. Bottom: box plot showing myCAF spatial cross-correlation with PTC and pEMT-PTC across 12 PTC samples containing PTC and pEMT-PTC cells. *P* value calculated with paired *t* test. (**F**) Representative LAMB3 IHC at the leading edge of PTC. (**G**) Spatial ligand-receptor interaction weights in PTC. Left: labeling of spatial barcodes by the population with the highest RCTD score in representative PTC Thy7. Right: spatial ligand-receptor interaction weights between labeled populations on the left (line widths indicate interaction weights). Bottom: Box plot showing myCAF interaction weights with PTC and pEMT-PTC across 4 PTCs with sufficient pEMT-PTC spatial barcodes for ligand-receptor interaction analysis. *P* value calculated with Wilcoxon signed-rank test. (**H**) Heatmap showing the top 10 signaling patterns in PTC Thy7. Spatial barcode populations at bottom. (**I**) UMAP of ATC tumor cell subclustering colored by pEMT scores for 2 ATCs with pEMT tumor cell populations. (**J**) Spatial feature plots of Thy4 with RCTD scores generated by replacing tumor cell clusters with Lu et al. ATC09 subclustering. (**K**) Box plot showing myCAF spatial cross-correlation with ATC and pEMT-ATC across 7 *BRAF*^V600E^ ATC samples with pEMT and myCAF populations. *P* value calculated with paired *t* test. (**L**) Representative LAMB3 IHC in *BRAF*^V600E^ ATC.

CAFs are known to modulate tumor cell behavior to support cellular invasion. Our group recently discovered a CAF-adjacent leading-edge tumor cell population, found in *BRAF*^V600E^ ATC with epithelioid/squamous morphology, as well as in invasive PTC. The leading-edge tumor cells showed strong expression of *TNC* (45) and striking phenotypic resemblance to a laminin subunit beta 3–positive (LAMB3^+^) TNC^+^ partial epithelial-mesenchymal transition (pEMT) phenotype described in head and neck squamous cell carcinoma (46). Thus, we investigated a potential interaction between invasive pEMT cells and myCAFs in PTC and ATC. Subclustering PTC cells identified a population with enrichment of the pEMT signature from Puram et al. (46) and increased interactions with myCAFs (*P* = 0.015; Figure 11, A–C, and Supplemental Figure 11, A and B). Spatial mapping of pEMT-PTC cells across pediatric and adult PTCs, as well as PTCs dedifferentiating into ATCs, highlighted a *LAMB3*^+^ pEMT invasive cellular population directly abutting myCAFs, which we further confirmed with IHC for LAMB3 (Figure 11, D–F, and Supplemental Figure 11C). We performed spatial ligand-receptor interaction analysis across 4 PTCs with robust pEMT-PTC populations. All 4 showed increased interaction between pEMT-PTC cells and myCAFs relative to non-pEMT-PTC cells (*P* = 0.13). The top upregulated signaling pathways included collagen, laminin, and tenascin, consistent with our recent work highlighting interaction between tumor-derived tenascin-c and myCAF-derived WNT ligands that drives tumor invasion (Figure 11, G and H, and Supplemental Figure 11, D–F) (45).

**Figure 12:**
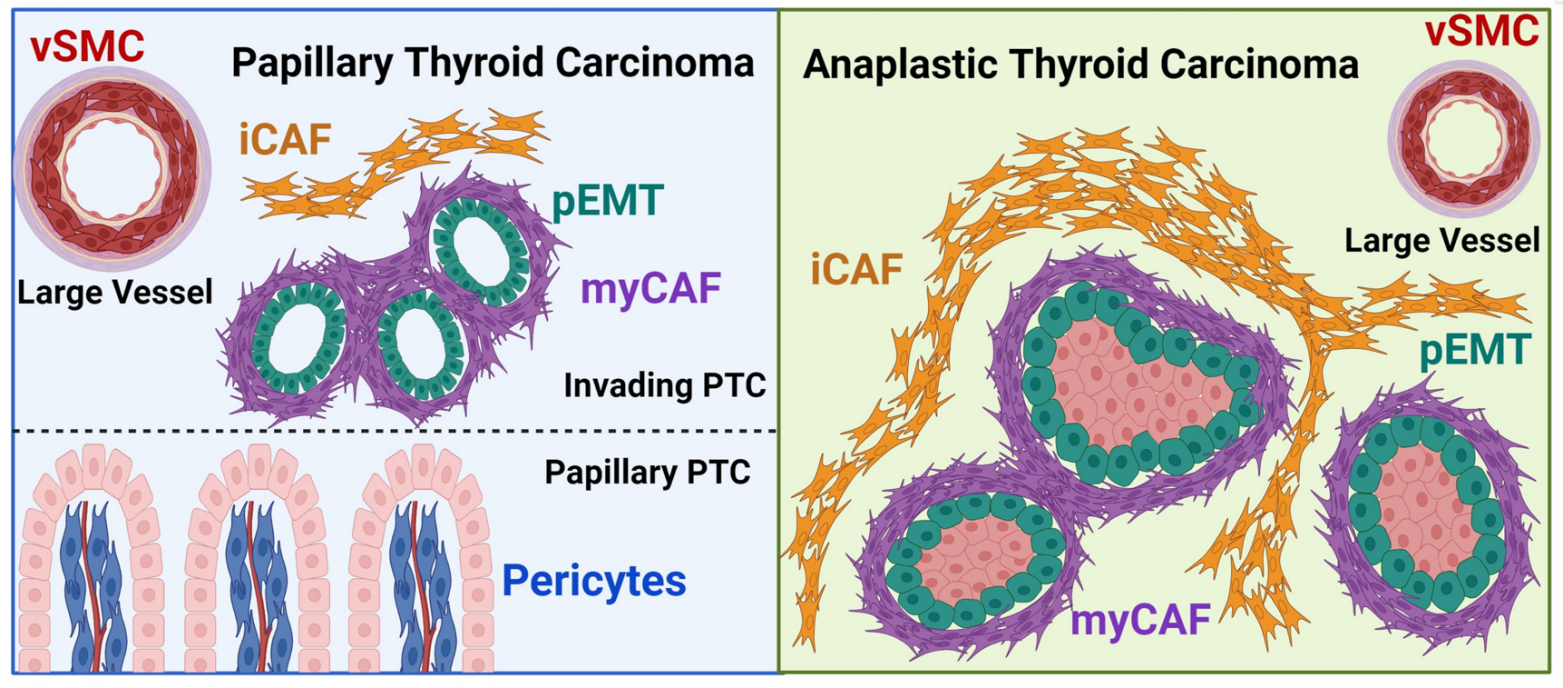
Overview of stromal architecture in thyroid cancer progression.

Because ATC tumors subcluster by sample, we looked for pEMT populations within individual *BRAF*^V600E^ tumor samples. In 2 ATCs we identified a subset of tumor cells with enrichment of pEMT genes and increased interaction with myCAFs (Figure 11I and Supplemental Figure 11, G and H). Spatial mapping of ATC and pEMT-ATC tumor cells highlighted a pEMT leading-edge phenotype intermixed with myCAFs in *BRAF*^V600E^ ATCs (Figure 11, J and K). IHC for LAMB3 further confirmed a myCAF-adjacent leading-edge pEMT phenotype (Figure 11L). The pEMT gene signature used to identify leading-edge cells was associated with aggressive tumor phenotypes across bulk RNA-sequencing cohorts (Supplemental Figure 12). Together, we show tumor-adjacent myCAFs directly communicating with invasive, pEMT-like tumor cells in both aggressive PTCs and ATCs (Figure 12).

## Discussion

Given the mounting evidence that CAFs shape tumor behavior and immunity across cancers, there is interest in therapeutic targeting of CAFs for treatment of highly lethal ATC (25, 27, 39, 47–51). However, pan-fibroblast-targeting strategies are unlikely to be effective (4–6), and specific CAF subset modulation in thyroid cancer is hindered by the lack of comprehensive profiling of subtypes and spatial localization. To discover CAF heterogeneity and function in thyroid cancer, we created a thyroid cancer single-cell sequencing atlas (26–32) and spatially resolved CAF dynamics in invasive tumors at sites of dedifferentiation from papillary to anaplastic morphology. Through this work, we identify CAF subsets that serve as critical mediators of cancer progression, inflammatory thyroiditis, and tumor hemodynamics. myCAFs in thyroid cancer are centrally located partners in invasion, directly abutting and communicating with invading tumor cells to alter extracellular matrix and differentiation programs. In contrast, iCAFs are tumor distant and enriched in areas of lymphocytic thyroiditis, supporting recent work showing fibroblasts with CAF phenotypes in non-neoplastic conditions (36). We also distinguish a population of vSMCs that regulate large vessel hemodynamics in thyroid tumors. Finally, we show that pericytes localize within the tumor fibrovascular cores, likely regulating hemodynamics locally within tumor papillae. Additional research is needed to explore the potential of pericytes to serve as CAF precursors, given the similarities in gene expression between myCAFs and pericytes in thyroid cancer.

Spatial profiling of CAF-tumor interactions in thyroid cancer has numerous implications for patient care. Using multiple large patient cohorts across institutions, we confirm that myCAFs are associated with disease aggression and worse outcomes in thyroid cancer. Clinical diagnostic assays to detect myCAFs in thyroid cancer could identify high-risk patients and improve thyroid cancer prognostication. Additionally, targeting the direct interactions between myCAFs and invading tumor cells may represent a promising therapeutic strategy for invasive disease. Targeting of iCAFs in thyroid cancer may help increase patient response to immunotherapy. For patients with inflammatory autoimmune thyroid conditions, the modulation of iCAFs may serve to reduce thyroid destruction and improve functionality. Targeting of pericyte populations in thyroid cancer could represent a promising strategy for altering tumor hemodynamics and reducing tumor growth and vascularization. Finally, an understanding of the stromal organization of ATC based on our proposed classification as either epithelial or mesenchymal may allow for improved and personalized therapeutic decision-making in this highly lethal disease.

The primary limitation of our study is the resolution of the Visium sequencing platform without matched single-cell sequencing. While our single-cell atlas mitigates this limitation, additional large studies with paired samples are needed to better spatially characterize epithelial and mesenchymal ATCs. A second limitation is that ATC single-cell samples have limited numbers of tumor cells due to necrosis and low tumor purity. Finally, recent studies have indicated that *BRAF*^V600E^ ATCs may have better response to immunotherapy combined with targeted therapies than *BRAF* WT ATCs (52). Because our cohorts are retrospective and did not receive immunotherapy, we are not able to assess the impact of ATC subtyping into epithelial and mesenchymal categories on the response to immunotherapy and targeted therapy. Future work is needed to understand how targeting the robust stroma in epithelial ATCs impacts their response to therapy.

In conclusion, our findings elucidate tumor-CAF interactions spatially across thyroid cancer progression using a combination of single-cell, spatial, and bulk RNA-sequencing cohorts. We identify CAF populations important for thyroid physiology, with myCAFs specifically serving as critical modulators of invasion in thyroid cancer. The dynamic changes in CAFs as thyroid cancer dedifferentiates have broad implications across solid tumors, where the identification of tumor-supporting tumor-stroma interactions could revolutionize understanding of tumor biology and lead to novel CAF-targeting strategies.

## Methods

### Sex as a biological variable

Our study investigated single-cell and spatial transcriptomic sequencing data from male and female patients with thyroid cancer. Due to the rarity of ATC, samples from male and female patients were integrated and analyzed together.

### Generation of thyroid cancer single-cell atlas

#### Sample pre-processing and individual analysis.

Raw count matrices and metadata were downloaded from 7 published thyroid cancer single-cell RNA-sequencing datasets (26–32). R package Seurat 4.4.0 was used throughout pre-processing and atlas integration (34). For each sample, Seurat objects were generated and pre-processed to exclude cells with fewer than 200 unique features, fewer than 500 total counts, or greater than 10% mitochondrial reads. Doublet detection was run using R package scDblFinder 1.14.0 with default arguments and predicted doublets were removed (53). Sample NORM21 from Lu et al. was excluded from further analysis due to fewer than 200 cells passing quality control. Sample PTC10_T from Pu et al. was excluded due to concern over ambient RNA limiting clear separation of tumor and stromal clusters on independent sample analysis. Following pre-processing, each sample was normalized and variance stabilized using SCTransform with vst.flavor set to “v2” (54, 55). Normalized counts were annotated with human primary cell atlas (HPCA) labels using SingleR (56). Copy number inference was performed using R package CopyKAT1.1.0 using default settings with normalized counts as input to identify aneuploid cells (44). Samples were individually clustered and annotated with broad cell type labels (malignant, non-malignant thyrocyte, stromal, myeloid, NKT, endothelial, and B/plasma cell) based on HPCA labels, presence or absence of aneuploid cells, provided cell type labels from each sample’s reference paper when available, and expression of canonical marker genes. Broad cell type and HPCA labels were added to sample metadata prior to integration.

#### Sample integration and broad cell type labeling.

Samples were integrated using fast mutual nearest neighbors within R package SeuratWrappers 0.2.0 (57). First, raw RNA counts and metadata were merged from all individual samples. To limit over batch correcting biologically distinct cell types, samples were merged in order of heterogeneity of cell populations. Tumors containing PTC cells, ATC cells, and diverse microenvironment populations were merged first, and tumors with few microenvironment populations were merged later. The merged data were normalized and integrated with SeuratWrappers command RunFastMNN based on the top 3,000 variable features. The top 30 dimensions within the MNN integration were used for Seurat commands RunUMAP, FindNeighbors, and FindClusters. Broad clusters were determined at a resolution of 0.6 and visualized with uniform manifold approximation and projection (UMAP). Broad clusters were labeled based on consensus of HPCA labels, canonical marker genes, provided cell type labels from reference papers, and cell type metadata from individual sample analysis. Because the stromal and ATC clusters overlapped, each cell within this group was assigned as stromal or ATC based on the metadata from individual sample analysis. Within analysis of individual samples, stromal clusters and ATC clusters were distinct and differentiated by aneuploidy profiles. The identity of thyrocyte and tumor cell populations was further validated by generating module scores in Seurat with published TCGA PTC gene lists for thyroid differentiation, *BRAF*-like thyroid tumors, and *RAS*-like tumors, and a 12-gene panel for distinguishing ATC from PTC published by Han et al. (29, 33).

#### Stromal subclustering.

Stromal cell labels were extracted from the broad labels of the integrated thyroid atlas and used to subset raw RNA counts and metadata from the merged atlas. The subset RNA count data were integrated and clustered as described above in sample integration at a resolution of 0.2. Differential gene expression between each stromal subcluster and all other stromal cells was calculated using Seurat function FindMarkers with the test specified as “MAST” (58). Results were plotted with Seurat function DoHeatmap for the top 15 genes from each subcluster with expression in at least 50% of cells based on descending fold-change. Genes with log_2_ fold-change of at least 1 were included as marker genes for each cluster (Supplemental Table 2) and used for gene ontology analysis. Additionally, pathway activity scores were generated for each stromal cell using R package PROGENy 1.22.0 (59, 60) and summarized across stromal subclusters following the analysis pipeline from Figure 3 of Gao et al. (https://github.com/aliceygao/pan-Fibroblast) (7). The identity of stromal subclusters was informed by generating module scores in Seurat with published CAF subtype and perivascular gene lists (8–15, 35, 36). Module scores were plotted using Seurat function DotPlot. Module scores and marker genes were also mapped onto the stromal UMAP using Seurat function FeaturePlot.

#### Pseudotime analysis of stromal cell subtypes.

Pseudotime analysis was performed using Monocle3 v1.4.26 and the partition-based graph abstraction (PAGA) framework in Scanpy v1.11.3 (61–63). For Monocle3, a cell_data_set was constructed from Seurat-derived expression and metadata with direct incorporation of batch-corrected MNN components and UMAP embeddings from the Seurat single-cell atlas object. Trajectory inference was carried out using Monocle3’s graph learning, and pseudotime was ordered from a manually selected root node. In parallel, PAGA was applied in Scanpy using MNN-based neighborhood graphs and Leiden clustering. Diffusion pseudotime was computed from a root cell in the vSMC cluster.

#### Differential abundance testing.

Differential abundance testing of stromal subclusters and epithelial clusters was performed with R package MiloR 1.8.1 (https://github.com/MarioniLab/miloR) (64). For buildGraph and makeNhoods functions, parameters k and d were both set to 30% and 10% of cells were sampled for makeNhoods. Results were plotted with function plotDAbeeswarm. Neighborhoods with a spatial false discovery rate (FDR) of less than 0.1 were colored corresponding to the condition they were enriched in.

#### Ligand-receptor interaction analysis.

Ligand-receptor interaction analysis was performed for individual samples within the integrated atlas with R package CellChat 2.1.2 using published pipelines (65, 66). Communication probabilities were calculated using triMean. Interaction weights between myCAFs and tumor cell populations were compared for samples containing at least 100 pEMT-PTC and 100 non-pEMT-PTC tumor cells. Interaction weights in Hashimoto thyroiditis paratumor samples were calculated for cell populations with at least 10 cells, excluding PTC. Interaction weights between cell populations were plotted with CellChat function netVisual_circle. The top 10 signaling pathways were plotted with CellChat function netAnalysis_signalingRole_heatmap.

### Bulk RNA-sequencing analysis

#### Sequencing cohorts.

Stromal and tumor cell subpopulation abundance and disease associations were analyzed in 5 thyroid cancer bulk RNA-sequencing cohorts: the VUMC/UW cohort (312 samples spanning benign and malignant neoplasms), TCGA (496 PTCs), CNUH/SNUH cohort (263 normal thyroids, 348 PTCs, 5 PDTCs, 16 ATCs), MD Anderson (108 PTCs), and St. Jude Children’s Research Hospital (46 PTCs) (31, 33). Sequencing and processing of the VUMC/UW cohort was previously described (25). For this study, raw counts were transcripts per million (TPM) normalized. TCGA data were downloaded from cBioPortal For Cancer Genomics (https://www.cbioportal.org), including previously calculated *BRAF*-*RAS* scores, American Thyroid Association risk stratification, and metastasis data (33, 43, 67, 68). TCGA PTC fibrosis scores from previously published imaging analysis were associated with stromal subpopulation abundance (39). The CNUH/SNUH cohort was downloaded from National Center for Biotechnology Information (NCBI) Gene Expression Omnibus (GEO) (GSE213647) and TPM normalized. The 108 PTCs in the MD Anderson cohort were processed and sequenced using previously described methods (69). Raw counts were TPM normalized. The St. Jude cohort was downloaded from the St. Jude Cloud (https://www.stjude.cloud/), and raw counts were TPM normalized for GSVA analysis or DESeq2 normalized for BRS calculation (70).

#### Calculating gene expression scores.

Gene expression scores for all bulk RNA-sequencing cohorts were generated via single-sample GSVA with R package GSVA 1.48.3 using default arguments (71). GSVA scores were generated for each stromal subcluster marker gene list (Supplemental Table 2), Puram et al. published pEMT marker genes, and our previously published 549-gene thyroid cancer MAP score (25, 46). For the MD Anderson, VUMC/UW, and CNUH/SNUH cohorts, TPM-normalized counts were used as input. For TCGA cohort, RSEM-normalized counts were used as input. BRS was previously calculated for TCGA and VUMC/UW cohorts (25, 33). BRS was calculated for the St. Jude cohort as previously described (25).

#### Survival analysis.

A total of 108 PTCs from the MD Anderson and 150 malignant samples from the VUMC/UW cohort had clinical follow-up data for PFS and OS analysis. These cohorts were split into 2 groups by 50th percentile for stromal and tumor subpopulation scores of interest, and survival analysis was performed as previously described (25). Kaplan-Meier survival curves were compared using the log-rank test. Cox proportional hazards models were also calculated for stromal and tumor subpopulation scores and summarized with forest plots. In the forest plots, the hazard ratio with a 95% confidence interval was reported for an IQR increase when scores were analyzed as continuous variables.

### Spatial transcriptomics analysis

#### Platform.

The Visium Spatial Gene Expression v1 for formalin-fixed, paraffin-embedded (FFPE) tissue platform was used to generate spatial transcriptomics data (10x Genomics).

#### FFPE block selection and sequencing.

Eight ATC FFPE blocks were previously processed by our lab (Supplemental Figure 7 samples Thy2-4, Thy8, Thy10, Thy14, Thy19, and Thy20) (25). An additional 20 FFPE blocks, including 8 pediatric PTCs, 5 adult PTCs, and 7 ATCs, were processed. Tumor regions were chosen by pathologist review to spatially capture areas of progression from PTC to ATC and areas with mixed tumor and stroma. One of the previously processed ATCs (Thy10) had mixed PTC/ATC histology, and 6 of the newly processed ATCs contained PTC components.

#### Sample processing and sequencing.

FFPE tissue sections, 5 μm were cut onto Visium Gene Expression Slides (Visium Spatial Gene Expression Slide Kit, PN-1000188). Slides were subsequently stained, imaged, processed, and sequenced as previously described (25). The Visium Human Transcriptome Probe Set v1.0 was used, and sequencing was performed using the NovaSeq 6000 platform (Illumina).

#### Data processing.

Visium sequencing FASTQ files and original magnification, 20×, image scans were pre-processed with Space Ranger 2.0.0 (10x Genomics). Space Ranger outputs were loaded into R package Seurat 4.4.0 using function Load10X_Spatial or R package SingleCellExperiment 1.22.0 using function readVisium (34, 72). Subsequent analyses were performed with either Seurat objects or SingleCellExperiment objects depending on the compatibility of the published analysis method. Spatial sequencing reads were normalized and variance stabilized using Seurat function SCTransform with vst.flavor set to “v2” (54, 55).

#### Enhanced resolution gene expression plots.

Individual genes were plotted at subspot resolution using BayesSpace 1.10.1 following the pipeline in the package vignette (https://github.com/edward130603/BayesSpace) (73).

#### Cell type deconvolution of spatial barcodes.

Raw RNA counts and broad cluster labels from the integrated thyroid cancer single-cell RNA-sequencing atlas were used as a reference for deconvolution of spatial transcriptomics data. The broad stromal cluster labels were split into iCAF, myCAF, APOE^+^ PVL, pericyte, and vSMC subpopulations based on stromal subclustering. Deconvolution was repeated with the PTC cluster split into PTC and pEMT-PTC based on PTC subclustering results and with the tumor cells subset to only ATC09 from Lu et al. with ATC and pEMT-ATC distinction. Deconvolution was performed with R package spacexr 2.2.1 using the Robust Cell Type Decomposition (RCTD) algorithm following the pipeline for Visium samples (https://github.com/dmcable/spacexr) (74). The run.RCTD function was run with doublet_mode set to “full.” Determining malignant cell fraction of spatial barcodes in *BRAF* WT ATCs was performed with R package SpaCET 1.2.0 (https://github.com/data2intelligence/SpaCET) with cancer type set to “PANCAN” (75).

#### Spatial cross-correlation.

Spatial cross-correlation analysis between RCTD scores was performed with R package MERINGUE 1.0 (https://github.com/JEFworks-Lab/MERINGUE) (76). Visium barcode coordinates were extracted from Seurat objects using the Seurat command GetTissueCoordinates with default arguments. Spatial neighbors were calculated by inputting barcode coordinates to the MERINGUE function getSpatialNeighbors with filter distance set to 7.5. Spatial neighbors and a matrix of normalized RCTD score weights were input into MERINGUE function spatialCrossCorMatrix to calculate spatial cross-correlation statistics between deconvoluted cell populations. The resulting spatial cross-correlation matrix was plotted with R package corrplot 0.92 using the corrplot function with order set to “hclust.” Box plots of spatial cross-correlation between populations of interest were plotted across samples using ggplot2 3.5.0 (77).

#### Spatial ligand-receptor interaction analysis.

Spatial ligand-receptor interaction analysis was performed with R package CellChat 2.1.2 using the v2 human database subset to protein signaling cell-cell communication genes and pathways. Spatial barcode identities for ligand-receptor interaction analysis were assigned based on the cell type composing a majority of the barcode following RCTD. SCT-transformed expression data were used as input. Communication probabilities were computed using triMean, an interaction range of 250 μm, a contact-dependent interaction range of 100 μm, and a minimum number of Visium barcodes per group of 10, as recommended in CellChat tutorials (https://github.com/jinworks/CellChat) (65, 66).

### Tissue staining of FFPE tissue

#### Multiplex immunofluorescence.

Multiplex immunofluorescence staining of 33 ATC FFPE blocks (19 *BRAF* WT, 14 *BRAF*^V600E^) for FAP (Abcam ab207178 recombinant rabbit monoclonal anti-FAP IgG, clone EPR20021, 1:100, Abcam) and pan-cytokeratin (eBioscience 53-9003-82 mouse monoclonal anti-pan cytokeratin IgG1 AF488, clone AE1/AE3, 1:100, Thermo Fisher Scientific) was performed as previously described (25). See Supplemental Methods for additional details.

#### IHC.

Tissue sections, 5 μm were cut from FFPE blocks. Deparaffinization and antigen retrieval were performed as previously described (25), and tissues were stained with primary antibodies marking stromal subpopulations (Abcam ab5694 rabbit polyclonal anti-alpha smooth muscle actin IgG, 1:500; Abcam ab314670 rabbit recombinant monoclonal anti-RGS5 IgG, clone EPR28539-64, 1:500; Invitrogen PA5-21514 rabbit polyclonal anti-laminin beta-3 IgG, 1:100; Invitrogen PA5-34641 rabbit polyclonal anti-periostin IgG, 1:100; Sigma-Aldrich HPA040520 affinity isolated rabbit polyclonal anti-APOD IgG, 1:500). See Supplemental Methods for additional details.

### Statistics

All statistical tests are 2 sided unless otherwise specified. Specific statistical tests are specified in figure legends and in Methods. For bulk RNA-sequencing cohorts, continuous variables were summarized with box plots and tested for significance using Wilcoxon rank-sum test. Kruskal-Wallis test with subsequent pairwise Wilcoxon rank-sum tests with Bonferroni’s correction was used when comparing more than 2 groups. Box plot lines indicate quartiles, and the whiskers indicate 1.5× the IQR. All statistical tests used a significance threshold of *P* less than 0.05 except for differential abundance testing with MiloR, where an FDR of 0.1 was used. Statistical analyses were performed using R 4.3.1.

### Study approval

FFPE patient blocks were used for spatial transcriptomics, immunofluorescence, and IHC studies as approved by Vanderbilt University Medical Center Institutional Review Board 241741.

### Data availability

Numerical values for individual data points in plots throughout the paper are available in the Supporting Data Values file. Single-cell RNA-sequencing data sets were downloaded from publicly available databases (Gene Expression Omnibus accession codes: GSE184362, GSE193581, GSE232237, GSE182416, GSE191288; Genome Sequence Archive accession number: HRA000686, contact the corresponding authors of Luo et al. (26) for assistance; CNGB Nucleotide Sequence Archive of China National GeneBank accession number: CNP0004262). TCGA bulk RNA-sequencing data were downloaded from cBioPortal For Cancer Genomics (https://www.cbioportal.org). Bulk RNA-sequencing data from the CNUH/SNUH cohort was downloaded from Gene Expression Omnibus (accession number: GSE213647). The St. Jude cohort was downloaded from the St. Jude Cloud (https://www.stjude.cloud/). The VUMC/UW bulk RNA-sequencing data are a retrospective cohort with restrictions on data sharing, as previously described (25). The spatial transcriptomics data have the same restrictions, and the IRB has requested that we do not publicly share individual-level sequencing data. Aggregate-level data for bulk RNA sequencing and spatial transcriptomics will be shared by lead contact upon request. Individual-level data are only available through collaboration following approval of the lead contact and the VUMC IRB. MD Anderson bulk RNA-sequencing data are uploaded to Gene Expression Omnibus (accession number: GSE310793). All code for reproducing analyses in the manuscript has been uploaded to GitHub: https://github.com/mloberg16/THCA_Fibroblast_Atlas, commit ID 9a70119.

## Author contributions

MAL conceptualized the study, performed experiments, analyzed the data, and wrote the manuscript. GJX and CJP prepared samples for sequencing. GJX and HCC performed data pre-processing and analysis. SCC performed statistical analyses. CC Wahoski, KPC, MLT, and DKW performed experiments. HAH conceptualized the study. JNG supervised clinical chart review. AAO, KAK, RGF, CC Wu, and MT performed data analysis. SMS, JLN, SLR, CCS, LAB, NB, BAM, ECH, and RHB collected clinical samples. JHC supervised chart review and reviewed clinical definitions. JRW supervised analysis of bulk RNA-sequencing data. QS and LTK performed data analysis and supervised computational analysis. LTK also performed sequencing experiments. EMJ supervised the study. KSL supervised computational data analysis. FY performed statistical analysis and supervised the study. EL supervised the study and conceptualized the design of the study. VLW supervised the study, conceptualized the design of the study, performed pathologist annotation and scoring, and wrote the manuscript. All authors reviewed and edited the manuscript.

## Funding support

This work is the result of NIH funding, in whole or in part, and is subject to the NIH Public Access Policy. Through acceptance of this federal funding, the NIH has been given a right to make the work publicly available in PubMed Central.

- American Society of Cytopathology (Young Investigator Award to VLW).
- American Thyroid Association (2019-0000000090 to VLW).
- NIH (R35GM122516, R01CA244188, and R01CA272875 to EL).
- NIH (VCORCDP K12CA090625, K08CA240901, and R01CA272875 to VLW).
- NIH F30CA281125 to MAL.
- NIH National Institute of General Medical Sciences T32GM007347 to the Vanderbilt MSTP supported MAL and MLT.
- NIH U01CA294527 and R01DK103831 to KSL.
- V Foundation for Cancer Research (Scholar Award to VLW).
- Children’s Cancer Research Fund (Research Award to VLW).
- American Cancer Society (133934-CSDG-19-216-01-TBG and RSG-22-084-01-MM to VLW.
- The Petrick Thyroid Cancer Research Fund to JRW.
- The Johns Hopkins Cancer Convergence Institute to EMJ and LTK.
- The Sidney Kimmel Cancer Center at Johns Hopkins to EMJ and LTK.
- EMJ is the Dana and Albert “Cubby” Broccoli Professor of Oncology.
- NIH to Vanderbilt Cell Imaging Shared Resources (CISR) core (5P30 CA68485-19, S10 OD023475-01A1, DK20593, DK58404, DK59637, and EY08126).

## Supplementary Material

Supplemental data

Supporting data values

## Version 1 01/09/2026

Electronic publication
