# Supplementary material for: An integrated single-cell and spatial transcriptomic atlas of thyroid cancer progression identifies prognostic fibroblast subpopulations
Source: JCI Insight. 2026 Jan 9;11(1):e191990. doi: 10.1172/jci.insight.191990 (PMC12890526; doi:10.1172/jci.insight.191990)
Supplement: Supplemental data [file jciinsight-11-191990-s035.pdf]

Supplemental Methods

Single-cell RNA-sequencing analysis supplemental methods

Tumor cell subclustering

ATC and PTC cell labels were independently extracted from broad labels of the integrated thyroid atlas and used to subset raw RNA counts and meta data from the merged atlas. The subset RNA count data were integrated and clustered as described above in sample integration at a resolution of 0.3. For ATC subclustering, differential gene expression analysis was performed between *BRAF* WT and *BRAF*^{V600E} cells. The top 10 marker genes with at least 80% expression in the population of interest were plotted with DoHeatmap in order of descending fold-change. For PTC subclustering, a pEMT module score was generated using a published pEMT gene signature from head and neck squamous cell carcinoma (46). PTC subclusters were labeled as “pEMT-PTC” or “PTC” based on their enrichment of the pEMT module score.

Spatial transcriptomic analysis supplemental methods

Annotation of spatial barcodes

Spatial barcodes were annotated using Loupe Browser 8.1.1 (10x Genomics, Pleasanton, CA) by a practicing pathologist with greater than 10 years of experience (VLW) and a pathologist trainee. In five samples (Peds02, Thy5, Thy11, Thy9, and Thy13), barcodes were assigned as within either a large perivascular area or tumor/other area. Barcodes from two representative PTCs (Thy15 and Thy17) were annotated as tumor or stroma. Within PTC regions of five PTCs (Thy15, Thy16, Thy17, Peds07, and Peds08), barcodes were further split into pure PTC barcodes and barcodes containing a mix of tumor cells and fibrovascular cells found at the central core of PTCs. Pathologist annotations were used to validate the localization of stromal cell deconvolution with RCTD (74).

Visium distance calculations

Visium barcode coordinates were extracted from Seurat objects using the Seurat command GetTissueCoordinates with scale set to NULL. To convert the coordinates from pixels to µm, the coordinates of each sample were multiplied by 65 (the approximate diameter in µm of each Visium spatial barcode) and divided by the full resolution spot diameter in pixels (generated by Space Ranger and located in the scalefactors_json.json file). Using the tissue coordinates in µm, Euclidean distance was calculated between individual coordinates. A distance matrix was generated for each sample containing sequencing barcodes as both rows and columns with values containing the distance between barcode pairs. For minimum distance calculations, distance matrices were subset to rows and columns containing spatial barcodes of interest. For example, the minimum distance for barcodes with at least 10% PTC normalized RCTD score weight from barcodes with at least 10% myCAF normalized RCTD score weight was calculated by subsetting the rows of the distance matrix to PTC barcodes and the columns to myCAF barcodes and returning the minimum distance value in each row. For analysis across samples, the average minimum distance was calculated for each sample of interest.

Tissue staining of FFPE tissue supplemental methods

Multiplex immunofluorescence

Multiplex immunofluorescence staining of 33 ATC FFPE blocks (19 *BRAF* WT, 14 *BRAF*^{V600E}) for fibroblast activating protein alpha (Abcam ab207178 recombinant rabbit monoclonal anti-fibroblast activation protein alpha (FAP) IgG, clone EPR20021, 1:100, Abcam, Cambridge, UK) and pan-cytokeratin (eBioscience 53-9003-82 mouse monoclonal anti-pan cytokeratin IgG1 AF488, clone AE1/AE3, 1:100, Thermo Fisher, Waltham, MA) was performed as previously described (25). Representative multiplex immunofluorescence images were scored by a practicing pathologist with greater than 10 years of experience and thyroid expertise (VLW). For each ATC tissue section, FAP staining of stromal cells was categorized as none, low, medium, or high, and FAP staining of tumor cells was categorized as none, low, or medium. Tumor cells were identified by nuclear morphology. An overall fibroblast pattern was assigned based on FAP staining of stromal cells as absent, intermixed, or tumor adjacent. Tumor cell

pan-cytokeratin was scored as none, low, medium, or high. The proportions for these staining categories were compared between *BRAF* WT and $BRAF^{V600E}$ ATCs using Fisher's exact test and plotted as bar plots using R package ggplot2 3.5.0 (77). The pancytokeratin and FAP staining scores were correlated with bulk RNA GSVA scores generated from the same tumors using Spearman's rank correlation coefficient. Correlations were calculated and plotted using R package corrplot 0.92.

Immunohistochemistry

5 µm tissue sections were cut from FFPE blocks. Deparaffinization and antigen retrieval were performed as previously described (25). Tissues were treated with BLOXALL endogenous blocking solution, peroxidase and alkaline phosphatase (SP-6000-100, Vector Laboratories, Newark, CA) for 10 min, washed with 0.05% Tween 20 in PBS, and blocked for 2 h with 10% goat serum in PBS (blocking buffer). Primary antibodies (Abcam ab5694 rabbit polyclonal anti-alpha smooth muscle actin IgG, 1:500; Abcam ab314670 rabbit recombinant monoclonal anti-RGS5 IgG, clone EPR28539-64, 1:500; Invitrogen PA5-21514 rabbit polyclonal anti-laminin beta-3 IgG, 1:100; Invitrogen PA5-34641 rabbit polyclonal anti-periostin IgG, 1:100; Sigma-Aldrich HPA040520 affinity isolated rabbit polyclonal anti-APOD IgG, 1:500) were diluted in blocking buffer and incubated on tissue sections at 4°C for 16 h (Abcam, Cambridge, UK; Thermo Fisher, Waltham, MA; MilliporeSigma, Burlington, MA). Tissue sections were washed with 0.05% Tween 20 in PBS and incubated for 30 minutes with ImmPRESS HRP horse anti-rabbit IgG polymer (MP-7801-15, Vector Laboratories, Newark, CA). Tissue sections were washed with 0.05% Tween 20 in PBS and developed via incubation for 2 min with an equal mix of reagent 1 and reagent 2 of ImmPACT DAB EqV Substrate (MP-7801-15, Vector Laboratories, Newark, CA). Following development, tissue sections were rinsed in distilled water, counterstained for 3 min with Mayer's Hematoxylin (MHS32-1L, MilliporeSigma, Burlington, MA), rinsed in tap water, washed for 1 min in Scott's water (10 g magnesium sulfate, 2g sodium bicarbonate, 1 L water), rinsed in tap water, dehydrated (1 min each of 25% ethanol, 50% Ethanol, 70% ethanol, 100% ethanol, and 3 min xylene), and cover slipped. APOD and POSTN staining of stromal cells were scored by a practicing pathologist with greater than 10 years of experience and thyroid expertise (VWL) as none (0), low (1), medium (2), or high (3) and tumor adjacent,

intermediate, or distant across 10 follicular nodular disease samples, 10 Hashimoto thyroiditis samples, 5 PTCs, 6 mixed PTC/ATC samples, and 5 ATCs.

Supplemental Tables and Figures

Supplemental Table 1: Single-cell atlas individual sample meta data.

Sample	Paper	*$BRAF^{V600E}$*	*RAS*	*TP53*	*TERT*p	*RET*/*FARP1*	Tissue type	Age	Sex	Hashimoto	Histology Subtype	Treatment	T Stage	N Stage	M Stage
Han_ATC34	Han	Mutation	No Mutation	U	Mutation	U	Primary Tumor	58	F	No	ATC	Naïve	U	U	U
Han_ATC35	Han	Mutation	No Mutation	U	Mutation	U	Primary Tumor	46	M	No	ATC: Spindled	Naïve	U	U	U
Han_ATC36	Han	Mutation	No Mutation	U	Mutation	U	Primary Tumor	68	F	No	ATC w/ Focal PTC	Naïve	U	U	U
Han_ATC37	Han	Mutation	No Mutation	U	Mutation	U	Primary Tumor	74	F	No	ATC	Famitinib/Camrelizumab	U	U	U
Lu_PTC01_T	Lu	U	U	U	U	U	Primary Tumor	33	F	U	PTC: Classical	Naïve	T2	N1b	n/a
Lu_PTC02_T	Lu	U	U	U	U	U	Primary Tumor	69	M	U	llicular-Variant w/ PDTC co	Naïve	T3b	Nx	Mx
Lu_PTC03_T	Lu	Mutation	No Mutation	No Mutation	Mutation	U	Primary Tumor	67	M	U	PTC: Tall-Cell	Dab-Tram	T4a	N1b	n/a
Lu_PTC03_P	Lu	n/a	n/a	n/a	n/a	n/a	Paratumor	67	M	U	PTC Paratumor	Dab-Tram	n/a	n/a	n/a
Lu_PTC04_T	Lu	No Mutation	No Mutation	No Mutation	No Mutation	U	Primary Tumor	15	F	U	PTC: Classical	Naïve	T4a	N1b	M1
Lu_PTC05_T	Lu	Mutation	No Mutation	No Mutation	No Mutation	U	Primary Tumor	23	F	U	PTC: Classical	Naïve	T4a	N1b	n/a
Lu_PTC06_T	Lu	Mutation	No Mutation	No Mutation	No Mutation	U	Primary Tumor	40	M	U	PTC: Classical	Naïve	T2a	N1b	Mx
Lu_PTC07_T	Lu	U	U	U	U	U	Primary Tumor	32	F	U	PTC: Classical	Naïve	T2	N0	n/a
Lu_PTC07_P	Lu	n/a	n/a	n/a	n/a	n/a	Paratumor	32	F	U	PTC Paratumor	Naïve	n/a	n/a	n/a
Lu_ATC08_T	Lu	Mutation	No Mutation	Mutation	No Mutation	U	Primary Tumor	70	F	U	ATC: Squamoid	zumab/vermuafenib/cobi	T4a	N1b	M1
Lu_ATC09_T	Lu	Mutation	No Mutation	No Mutation	Mutation	U	Primary Tumor	51	F	U	ATC: Spindled	Naïve	T4b	N1b	M1
Lu_ATC10_T	Lu	No Mutation	No Mutation	Mutation	No Mutation	U	Primary Tumor	68	M	U	ATC: Spindled	Naïve	T4a	N0	M0
Lu_ATC11_T	Lu	No Mutation	Mutation	No Mutation	Mutation	U	Primary Tumor	59	M	U	ATC: Spindled	Naïve	T4b	N1b	M1
Lu_ATC12_T	Lu	No Mutation	Mutation	Mutation	Mutation	U	Primary Tumor	51	F	U	ATC: Spindled	Naïve	T4a	N1b	M1
Lu_ATC13_T	Lu	No Mutation	Mutation	No Mutation	No Mutation	U	Primary Tumor	69	F	U	ATC: Spindled	Naïve	T3a	N0	M0
Lu_ATC14_T	Lu	No Mutation	No Mutation	Mutation	Mutation	U	Primary Tumor	59	M	U	ATC: Squamoid	Naïve	T3a	N0	M0
Lu_ATC15_T	Lu	U	U	U	U	U	Primary Tumor	48	F	U	ATC: Squamoid	Naïve	T4a	N0	M0
Lu_ATC17_T	Lu	No Mutation	Mutation	Mutation	Mutation	U	Primary Tumor	57	F	U	ATC: Spindled	Naïve	T4a	N0	M0
Lu_ATC18_T	Lu	U	U	U	U	U	Primary Tumor	65	F	U	ATC	Naïve	T3b	N1b	M1
Lu_ATC18_P	Lu	n/a	n/a	n/a	n/a	n/a	Paratumor	65	F	U	ATC Paratumor	Naïve	n/a	n/a	n/a
Lu_NORM19	Lu	n/a	n/a	n/a	n/a	n/a	Normal Thyroid	53	F	U	Normal	Naïve	n/a	n/a	n/a
Lu_PTC20_P	Lu	n/a	n/a	n/a	n/a	n/a	Paratumor	37	F	U	PTC Paratumor	Naïve	n/a	n/a	n/a
Pu_PTC01_T	Pu	Mutation	No Mutation	U	No Mutation	U	Primary Tumor	U	F	Yes	PTC: Classical	Naïve	T4a	N0	M0
Pu_PTC01_P	Pu	n/a	n/a	n/a	n/a	n/a	Paratumor	U	F	Yes	PTC Paratumor	Naïve	n/a	n/a	n/a
Pu_PTC02_T	Pu	Mutation	No Mutation	U	No Mutation	U	Primary Tumor	U	F	Yes	PTC: Classical	Naïve	T4a	N1b	M0
Pu_PTC02_P	Pu	n/a	n/a	n/a	n/a	n/a	Paratumor	U	F	Yes	PTC Paratumor	Naïve	n/a	n/a	n/a
Pu_PTC02_LLN	Pu	Mutation	No Mutation	U	No Mutation	U	Lymph Node	U	F	Yes	PTC: Classical	Naïve	T4a	N1b	M0
Pu_PTC03_T	Pu	No Mutation	No Mutation	U	No Mutation	U	Primary Tumor	U	M	No	PTC: Classical	Naïve	T1b	N1b	M0
Pu_PTC03_P	Pu	n/a	n/a	n/a	n/a	n/a	Paratumor	U	M	No	PTC Paratumor	Naïve	n/a	n/a	n/a
Pu_PTC03_LLN	Pu	No Mutation	No Mutation	U	No Mutation	U	Lymph Node	U	M	No	PTC: Classical	Naïve	T1b	N1b	M0
Pu_PTC03_RLN	Pu	No Mutation	No Mutation	U	No Mutation	U	Lymph Node	U	M	No	PTC: Classical	Naïve	T1b	N1b	M0
Pu_PTC04_SC	Pu	Mutation	No Mutation	U	Mutation	U	Subcutaneous Met	U	M	Yes	PTC: Classical	Prior Surgery + RAI	rT0	N0	M1
Pu_PTC05_T	Pu	No Mutation	No Mutation	U	No Mutation	U	Primary Tumor	15	M	No	PTC: Follicular-Variant	Naïve	T4a	N1b	M1
Pu_PTC05_P	Pu	n/a	n/a	n/a	n/a	n/a	Paratumor	15	M	No	PTC Paratumor	Naïve	n/a	n/a	n/a
Pu_PTC05_RLN	Pu	No Mutation	No Mutation	U	No Mutation	U	Lymph Node	15	M	No	PTC: Follicular-Variant	Naïve	T4a	N1b	M1
Pu_PTC06_RLN	Pu	Mutation	No Mutation	U	Mutation	U	Lymph Node	U	M	No	PTC: Tall-Cell	Prior Surgery	rT0	N1b	M0
Pu_PTC07_RLN	Pu	No Mutation	No Mutation	U	No Mutation	U	Lymph Node	U	M	No	PTC: Follicular-Variant	Prior Surgery	rT0	N1b	M0
Pu_PTC08_T	Pu	No Mutation	No Mutation	U	No Mutation	U	Primary Tumor	U	F	Yes	PTC: Classical	Naïve	T4a	N1b	M0
Pu_PTC08_P	Pu	n/a	n/a	n/a	n/a	n/a	Paratumor	U	F	Yes	PTC Paratumor	Naïve	n/a	n/a	n/a
Pu_PTC09_T	Pu	Mutation	No Mutation	U	No Mutation	U	Primary Tumor	U	F	No	PTC: Classical	Naïve	T1b	N1a	M0
Pu_PTC09_P	Pu	n/a	n/a	n/a	n/a	n/a	Paratumor	U	F	No	PTC Paratumor	Naïve	n/a	n/a	n/a
Pu_PTC10_RLN	Pu	Mutation	No Mutation	U	No Mutation	U	Lymph Node	U	M	No	PTC: Classical	Naïve	T4a	N1b	M1
Pu_PTC11_RLN	Pu	Mutation	No Mutation	U	No Mutation	U	Lymph Node	U	F	No	PTC: Classical	Prior Surgery + RAI	rT0	N1b	M1
Pu_PTC11_SC	Pu	Mutation	No Mutation	U	No Mutation	U	Subcutaneous Met	U	F	No	PTC: Classical	Prior Surgery + RAI	rT0	N1b	M1
Luo_ATC_WYF	Luo	No Mutation	No Mutation	No Mutation	No Mutation	U	Primary Tumor CNB	78	F	Yes	ATC	U	T4b	N1a	M1
Luo_ATC_MSQ	Luo	No Mutation	No Mutation	No Mutation	No Mutation	U	Primary Tumor CNB	78	F	Yes	ATC	U	T4a	N1a	M0
Luo_ATC_LJ	Luo	Mutation	No Mutation	No Mutation	No Mutation	U	Primary Tumor CNB	69	M	No	ATC	U	T4b	N1b	M0
Luo_PTC_XTZ	Luo	No Mutation	No Mutation	No Mutation	No Mutation	U	Primary Tumor	66	F	Yes	PTC	U	T3a	N1b	M0
Luo_Para_XTZ	Luo	n/a	n/a	n/a	n/a	n/a	Paratumor	66	F	Yes	PTC Paratumor	U	n/a	n/a	n/a
Luo_PTC_XYH	Luo	No Mutation	No Mutation	No Mutation	No Mutation	U	Primary Tumor	64	F	No	PTC	U	T1a	N0	M0
Luo_PTC_WJL	Luo	Mutation	No Mutation	No Mutation	No Mutation	U	Primary Tumor	56	M	No	PTC	U	T1b	N1b	M0
Lee_PT3	Lee	U	U	U	U	U	Primary Tumor	34	M	U	PTC	U	U	U	U
Lee_PT5	Lee	U	U	U	U	U	Primary Tumor	53	F	U	PTC	U	U	U	U
Lee_PT7	Lee	U	U	U	U	U	Primary Tumor	47	F	U	PTC	U	U	U	U
Lee_PT8	Lee	U	U	U	U	U	Primary Tumor	21	F	U	PTC	U	U	U	U
Lee_PT9	Lee	U	U	U	U	U	Primary Tumor	25	F	U	PTC	U	U	U	U
Lee_PT10	Lee	U	U	U	U	U	Primary Tumor	59	F	U	PTC	U	U	U	U
Lee_PT12	Lee	U	U	U	U	U	Primary Tumor	55	F	U	PTC	U	U	U	U
Lee_AT9	Lee	U	U	U	U	U	Primary Tumor	78	F	U	ATC	U	U	U	U
Lee_AT13	Lee	U	U	U	U	U	Primary Tumor	54	F	U	ATC	U	U	U	U
Lee_AT16	Lee	U	U	U	U	U	Primary Tumor	59	F	U	ATC	U	U	U	U
Lee_AT17	Lee	U	U	U	U	U	Primary Tumor	70	F	U	ATC	U	U	U	U
Lee_AT20	Lee	U	U	U	U	U	Primary Tumor	84	F	U	ATC	U	U	U	U
Hong_Thy01	Hong	n/a	n/a	n/a	n/a	n/a	Normal Thyroid	57	F	U	Normal Thyroid	U	n/a	n/a	n/a
Hong_Thy04	Hong	n/a	n/a	n/a	n/a	n/a	Paratumor	23	F	U	PTC Paratumor	U	n/a	n/a	n/a
Hong_Thy05	Hong	n/a	n/a	n/a	n/a	n/a	Paratumor	34	F	U	PTC Paratumor	U	n/a	n/a	n/a
Hong_Thy06	Hong	n/a	n/a	n/a	n/a	n/a	Paratumor	54	F	U	PTC Paratumor	U	n/a	n/a	n/a
Hong_Thy10	Hong	n/a	n/a	n/a	n/a	n/a	Paratumor	63	F	U	PTC Paratumor	U	n/a	n/a	n/a
Hong_Thy15	Hong	n/a	n/a	n/a	n/a	n/a	Paratumor	37	F	U	PTC Paratumor	U	n/a	n/a	n/a
Hong_N3-GEX	Hong	n/a	n/a	n/a	n/a	n/a	Paratumor	28	F	U	PTC Paratumor	U	n/a	n/a	n/a
Wang_T1L	Wang	No Mutation	U	U	U	Mutation	Primary Tumor	U	F	Yes	PTC: Classical	U	U	N0	U
Wang_T1R	Wang	Mutation	U	U	U	No Mutation	Primary Tumor	U	F	Yes	PTC: Classical	U	U	N0	U
Wang_T2L	Wang	Mutation	U	U	U	No Mutation	Primary Tumor	21-40	M	Yes	PTC: Classical/Follicular	U	U	N1b	U
Wang_T2R	Wang	Mutation	U	U	U	No Mutation	Primary Tumor	21-40	M	Yes	PTC: Classical/Follicular	U	U	N1b	U
Wang_T3L	Wang	Mutation	U	U	U	No Mutation	Primary Tumor	21-40	M	No	PTC: Classical	U	U	N1a	U
Wang_T3R	Wang	Mutation	U	U	U	No Mutation	Primary Tumor	21-40	M	No	PTC: Classical	U	U	N1a	U
Wang_NT	Wang	n/a	n/a	n/a	n/a	n/a	Normal Thyroid	0-20	F	No	Normal	U	n/a	n/a	n/a

U indicates unknown for samples with missing data
81 n/a indicates not applicable for non-malignant (paratumor or normal thyroid) samples

Supplemental Figure 1: Integrated single-cell atlas validation of tumor cell populations.

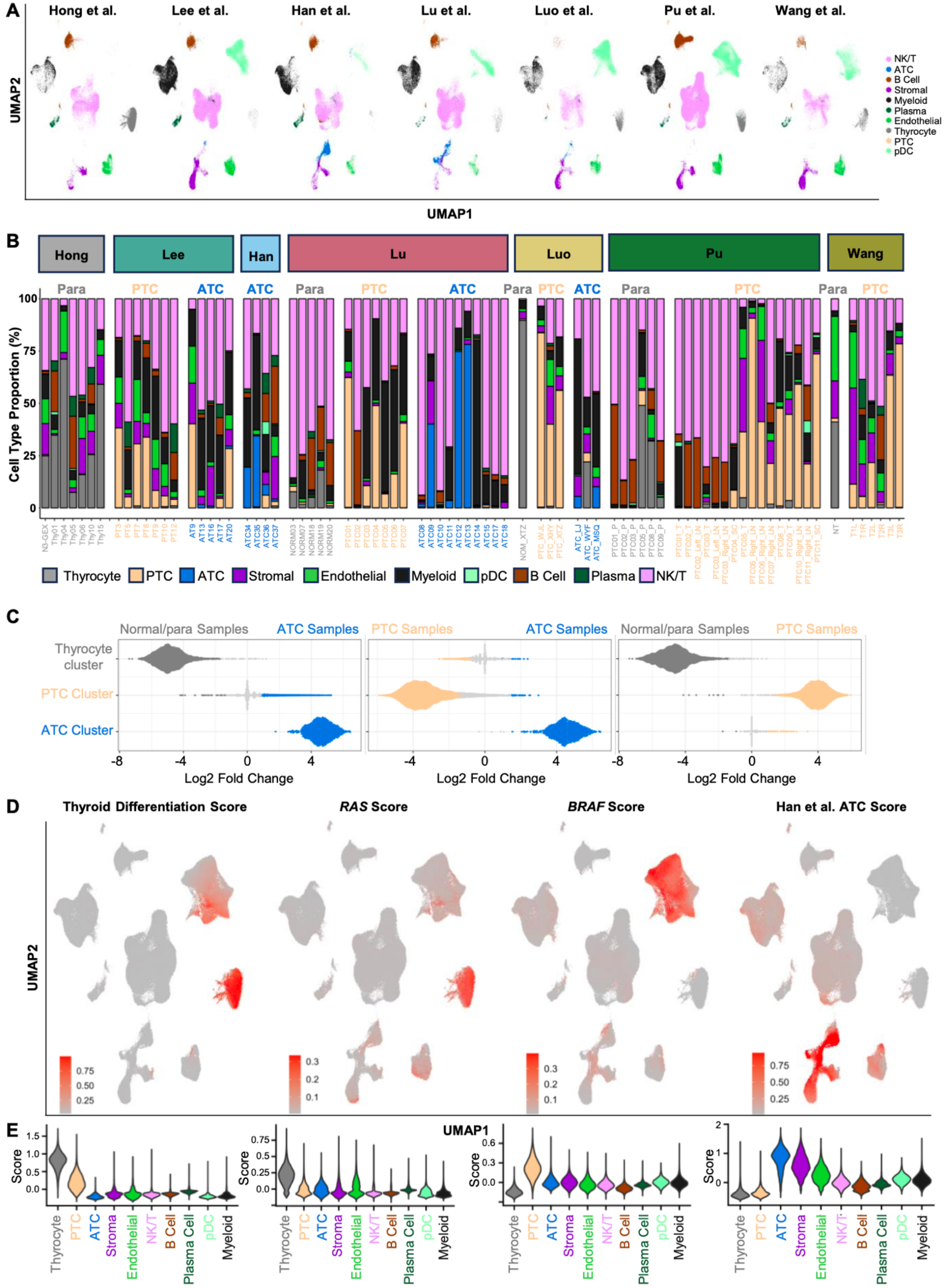

Supplemental Figure 1. Integrated single-cell atlas validation of tumor cell populations. (**A**) Broad cell population Uniform Manifold Approximation and Projection (UMAP) from **Figure 1B** split by paper. (**B**) Bar plots showing cell type proportion for each sample in the integrated thyroid cancer atlas. Papers and histologic subtype (anaplastic thyroid cancer, ATC; papillary thyroid cancer, PTC; normal/paratumor, Para) are labeled on top and sample names on bottom. (**C**) Milo differential abundance testing of broad epithelial clusters (thyrocyte, PTC, ATC) between normal/paratumor samples and ATC samples (left), PTC samples and ATC samples (middle), and normal/paratumor samples and PTC samples (right). Individual dots depict neighborhoods calculated by Milo. Coloring of individual neighborhoods as dark grey (normal/paratumor), light orange (PTC), or blue (ATC) indicates a spatial false discovery rate (FDR) of less than 0.1. Neighborhoods colored light grey have a spatial FDR greater than 0.1. (**D**) UMAP plot of integrated single-cell atlas showing module scores for thyroid differentiation, *RAS*-like genes, *BRAF*-like genes, and a 12 gene ATC score from Han et al. (left to right) (29, 33). (**E**) Violin plots for the module scores in **D** depicting scores by broad cluster identification. Abbreviations: pDC, plasmacytoid dendritic cell; NK/T, natural killer/T cell.

Supplemental Figure 2: Enriched genes and pathways in stromal subclusters.

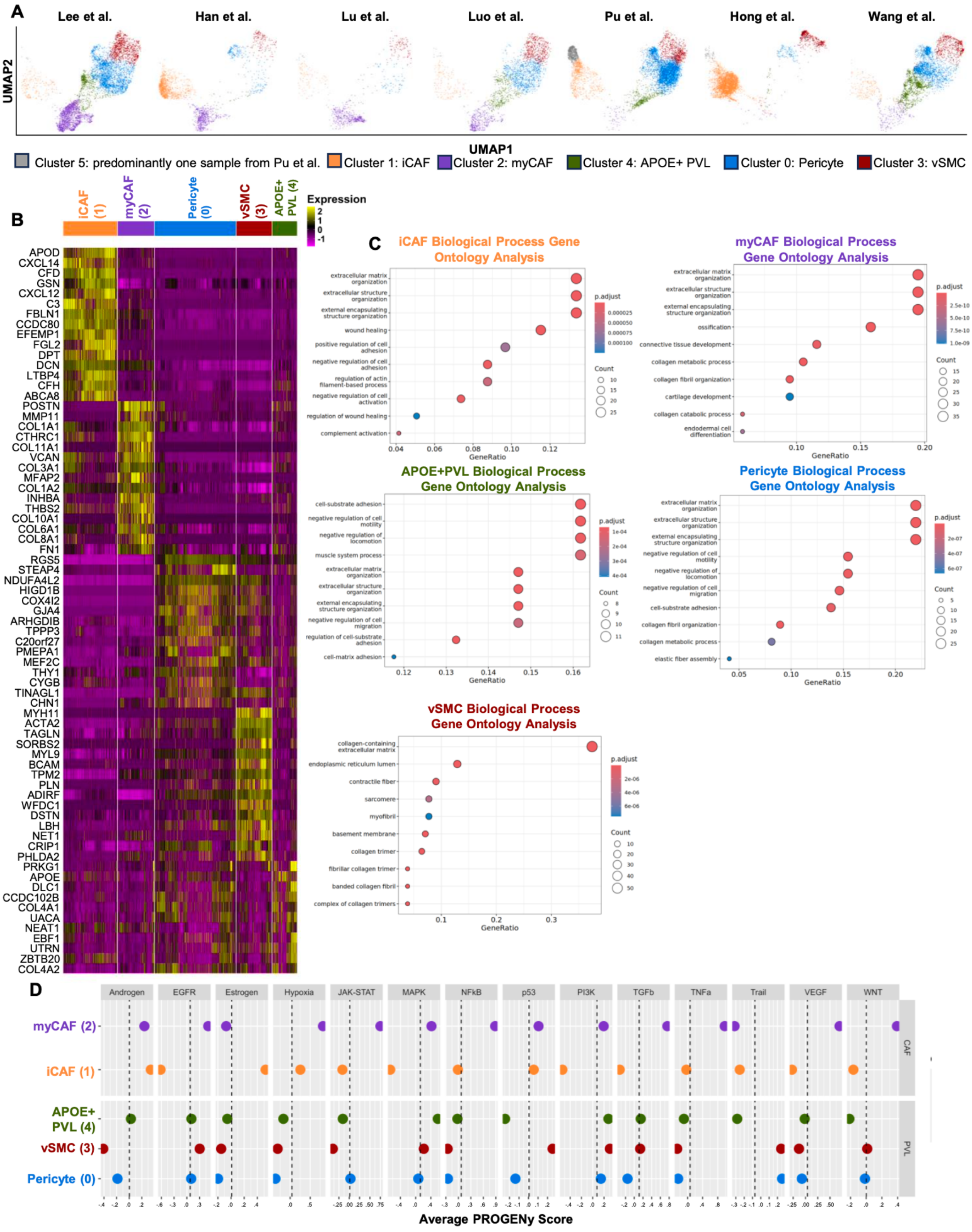

Supplemental Figure 2: Enriched genes and pathways in stromal subclusters. (**A**) Stromal cell subclustering Uniform Manifold Approximation and Projection (UMAP) from **Figure 2A** split by paper. (**B**) Heatmap showing scaled expression of the top 15 marker genes for each stromal subcluster with expression in at least 50 percent of cells in the cluster of interest. (**C**) Gene ontology analysis of the genes for each stromal subcluster with log2 fold-change enrichment of at least 1.0 (**Supplemental Table 2** genes) showing the top 10 biological processes for each cluster. (**D**) PROGENy pathway activity scores for each stromal subcluster.

Supplemental Table 2: Marker genes of CAF and PVL populations.

Cluster 2 (myCAF)

Genes	Log2FC	Genes	Log2FC
POSTN	3.41	ISLR	1.18
MMP11	3.25	MMP7	1.17
CST1	3.20	IFI6	1.16
COL1A1	2.86	SERPINE1	1.14
CTHRC1	2.77	H19	1.11
COL11A1	2.26	TMSB10	1.11
CXCL8	2.19	SLC16A3	1.10
VCAN	2.18	ANTXR1	1.08
COL3A1	2.11	LRRC15	1.08
MFAP2	2.02	EPSTI1	1.07
HSPA6	2.02	SAT1	1.07
COL1A2	2.02	CXCL10	1.06
INHBA	2.02	SPON2	1.06
THBS2	1.99	NNMT	1.06
COL10A1	1.97	RCN1	1.05
COL6A1	1.95	CALU	1.05
COL8A1	1.91	CYP1B1	1.05
FN1	1.90	P4HB	1.04
MMP14	1.89	ADAM12	1.04
MMP9	1.89	SOD2	1.03
SFRP2	1.88	LY6E	1.03
CTSK	1.88	NBL1	1.02
CXCL1	1.84	ENO1	1.02
COL6A3	1.83	ADAMTS2	1.01
COL5A2	1.78	CNN2	1.00
SULF1	1.70	RUNX2	1.00
MMP13	1.70		
CCN2	1.69		
LUM	1.68		
CCN1	1.65		
COL5A1	1.64		
AEBP1	1.63		
CTSB	1.56		
IL32	1.53		
IGF2	1.51		
RARRES2	1.51		
PLAU	1.50		
MMP1	1.49		
CDH11	1.46		
DIO2	1.45		
CD82	1.42		
FAP	1.42		
SPARC	1.39		
COL12A1	1.38		
SDC1	1.38		
COMP	1.37		
HTRA1	1.35		
COL6A2	1.32		
IFI27	1.31		
LGALS1	1.30		
ADM	1.30		
FTH1	1.29		
PLOD2	1.28		
SERPINH1	1.27		
MMP2	1.27		
TMEM158	1.26		
GJA1	1.25		
TNFRSF6B	1.25		
MXRA5	1.24		
PDPN	1.24		
TNFSF10	1.24		
MDK	1.24		
GREM1	1.24		
S100A16	1.23		
TNFAIP6	1.23		
PLAUR	1.22		
PKM	1.22		
RCN3	1.20		
CXCL6	1.19		
SPHK1	1.19		

Cluster 1 (iCAF)

Genes	Log2FC	Genes	Log2FC
APOD	4.57	SUCNR1	1.37
MYOC	3.78	S100A10	1.37
CXCL14	3.62	SLIT2	1.36
CFD	3.38	SCARA5	1.36
GSN	3.27	PTN	1.35
CXCL12	3.21	SSPN	1.34
PLA2G2A	3.14	FLRT2	1.33
C3	3.09	IGFBP3	1.33
FBLN1	3.07	PIK3R1	1.32
CCDC80	3.04	GAS1	1.30
EFEMP1	2.94	CDO1	1.30
FGL2	2.85	PID1	1.30
DPT	2.75	CPXM2	1.29
DCN	2.75	GDF10	1.29
LTBP4	2.68	CTSF	1.27
CFH	2.62	SVEP1	1.27
ADH1B	2.61	COL15A1	1.27
ABCA8	2.50	PLPP3	1.26
SRPX	2.50	LUM	1.26
MFAP4	2.49	HLA-DRB1	1.25
FBLN2	2.46	LSP1	1.25
C7	2.44	BOC	1.23
MGP	2.38	ANXA1	1.23
GPC3	2.36	AKR1C1	1.23
SFRP4	2.35	NFIA	1.22
ITM2A	2.35	COL14A1	1.22
CHRDL1	2.32	METTL7A	1.22
FBLN5	2.30	COLEC12	1.21
IGF1	2.28	ARL6IP5	1.21
PODN	2.22	HSPG2	1.20
IGFBP6	2.21	F10	1.20
SELENOP	2.19	FAM180B	1.20
OGN	2.17	CRISPLD1	1.20
ABI3BP	2.17	TMEM119	1.19
ANGPTL1	2.14	CD74	1.18
NOV	2.13	ABCA9	1.18
C1S	2.12	MGST1	1.18
CLU	2.10	WISP2	1.17
F3	2.02	ISLR	1.16
GPNMB	2.00	ELN	1.16
PRELP	1.98	SPRY1	1.15
PI16	1.94	SFRP1	1.15
SFRP2	1.93	ADD3	1.14
TNXB	1.89	PMP22	1.13
C1R	1.80	PSAP	1.13
TFF3	1.78	DHRS3	1.12
ZFP36L2	1.74	PLXDC2	1.12
LRP1	1.73	PLTP	1.12
CD34	1.72	HSPB6	1.12
ALDH1A1	1.72	RNASE4	1.11
MMP2	1.71	TSC22D3	1.11
OMD	1.68	LGALS3	1.10
LEPR	1.67	SERPING1	1.10
SERPINF1	1.66	ENG	1.10
FMO2	1.59	LAMA2	1.09
EMP1	1.57	PRNP	1.09
PTGDS	1.57	USP53	1.09
ABCA6	1.52	TSPAN8	1.07
ABCA10	1.50	ALDH2	1.06
CYBRD1	1.49	C16orf89	1.03
FGF7	1.48	NFIX	1.03
CST3	1.48	IGSF10	1.02
CCL4	1.48	TIMP3	1.02
PDGFRA	1.45	TG	1.01
SERPINE2	1.44	IGF2.1	1.01
CTGF	1.44	DIO3OS	1.01
PDGFRL	1.43	AHNAK	1.01
OSR1	1.42	TSHZ2	1.01
EPHX1	1.40	MATN2	1.00
NFIB	1.38		

Cluster 4 (APOE+ PVL)

Genes	Log2FC
PRKG1	2.33
APOE	2.30
SOX5	1.76
DLC1	1.59
PTPRG	1.36
PEAK1	1.30
PLCL1	1.28
CCDC102B	1.27
ARIH1	1.23
NRXN3	1.22
RASAL2	1.21
NRG3	1.20
PARD3	1.16
COL4A1	1.14
CACNA1C	1.13
APOC1	1.12
UACA	1.12
RBMS3	1.11
NEAT1	1.10
EBF1	1.09
UTRN	1.08
APBB2	1.06
ZSWIM6	1.06
ZBTB20	1.03
RNF152	1.01
CACNB2	1.01
COL4A2	1.01

Cluster 0 (Pericyte)

Genes	Log2FC
RGS5	2.58
FABP4	2.40
CD36	2.33
STEAP4	2.09
NDUFA4L2	1.89
HIGD1B	1.89
COX4I2	1.73
LHFP	1.66
FABP5	1.54
GJA4	1.49
ARHGDIB	1.43
TPPP3	1.38
C20orf27	1.35
PMEPA1	1.34
MEF2C	1.33
THY1	1.28
FAM162B	1.19
CYGB	1.17
TINAGL1	1.13
CHN1	1.12
FAM213A	1.11
MYO1B	1.08
KCNJ8	1.07
MARCKSL1	1.05
ARHGAP15	1.04
GMFG	1.02

Cluster 3 (vSMC)

Genes	Log2FC
MYH11	3.07
ACTA2	2.77
RERGL	2.67
MUSTN1	2.24
TAGLN	2.23
SORBS2	2.07
MYL9	2.05
BCAM	1.94
TPM2	1.93
PLN	1.88
ADIRF	1.83
WFDC1	1.80
DSTN	1.79
LBH	1.76
NET1	1.72
CRIP1	1.71
PHLDA2	1.67
MCAM	1.65
PPP1R14A	1.63
CSRP1	1.58
CASQ2	1.57
ACTG2	1.53
CDKN1A	1.52
CNN1	1.48
MYLK	1.41
RCAN2	1.38
C11orf96	1.32
CSRP2	1.31
GADD45B	1.24
MT1M	1.23
LMOD1	1.21
FAM107B	1.20
JAG1	1.19
CYCS	1.18
TPM1	1.17
NRARP	1.16
MFGE8	1.16
ADAMTS1	1.15
FLNA	1.15
FRZB	1.14
TINAGL1	1.13
CAV1	1.13
MAP3K20	1.12
NDUFA4	1.11
CRYAB	1.11
ACTN4	1.08
PTP4A3	1.05
MT1X	1.03
CRIP2	1.03
ID1	1.03
GPRC5C	1.02
BTG2	1.02
ATF3	1.01

Supplemental Figure 3: Stromal niches and subcluster trajectories.

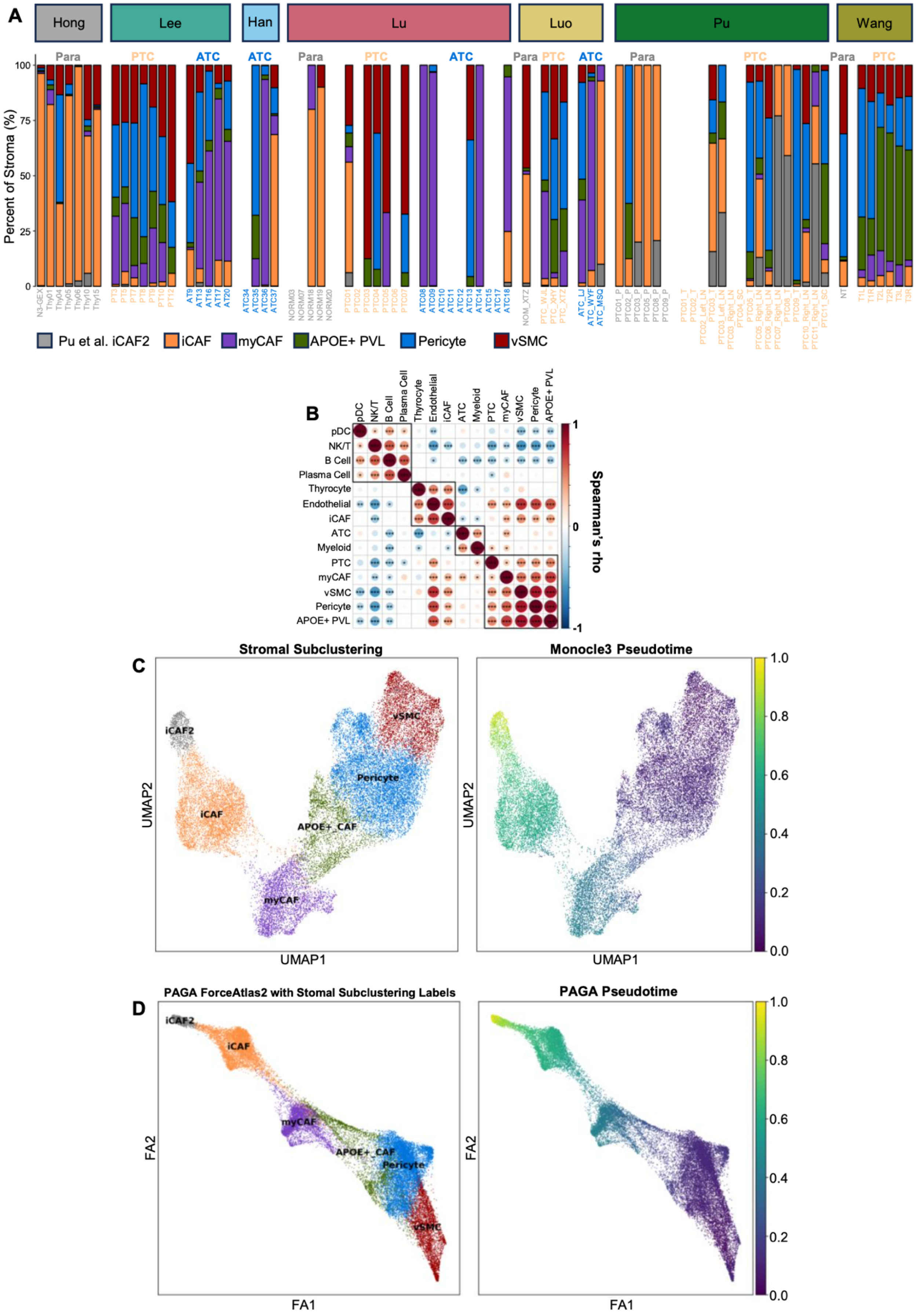

Supplemental Figure 3: Stromal niches and subcluster trajectories. (**A**) Bar plots showing composition of stromal cells in integrated thyroid cancer single-cell atlas for each individual sample. Samples with a blank space had no stromal cells identified. (**B**) Corrplot showing Spearman's rho correlations between the frequencies of stromal subcluster populations and the frequency of broader tumor and immune populations across all samples in the single-cell atlas. (**C** and **D**) Pseudotime trajectory analysis performed using (**B**) Monocle3 or (**C**) partition-based graph abstraction (PAGA). Dimensional reduction with CAF subcluster labels is displayed on the left with either Uniform Manifold Approximation and Projection (UMAP) or ForceAtlas2 (FA). Pseudotime is displayed on the right.

Supplemental Figure 4: Stromal cell abundance in bulk RNA-sequencing cohorts.

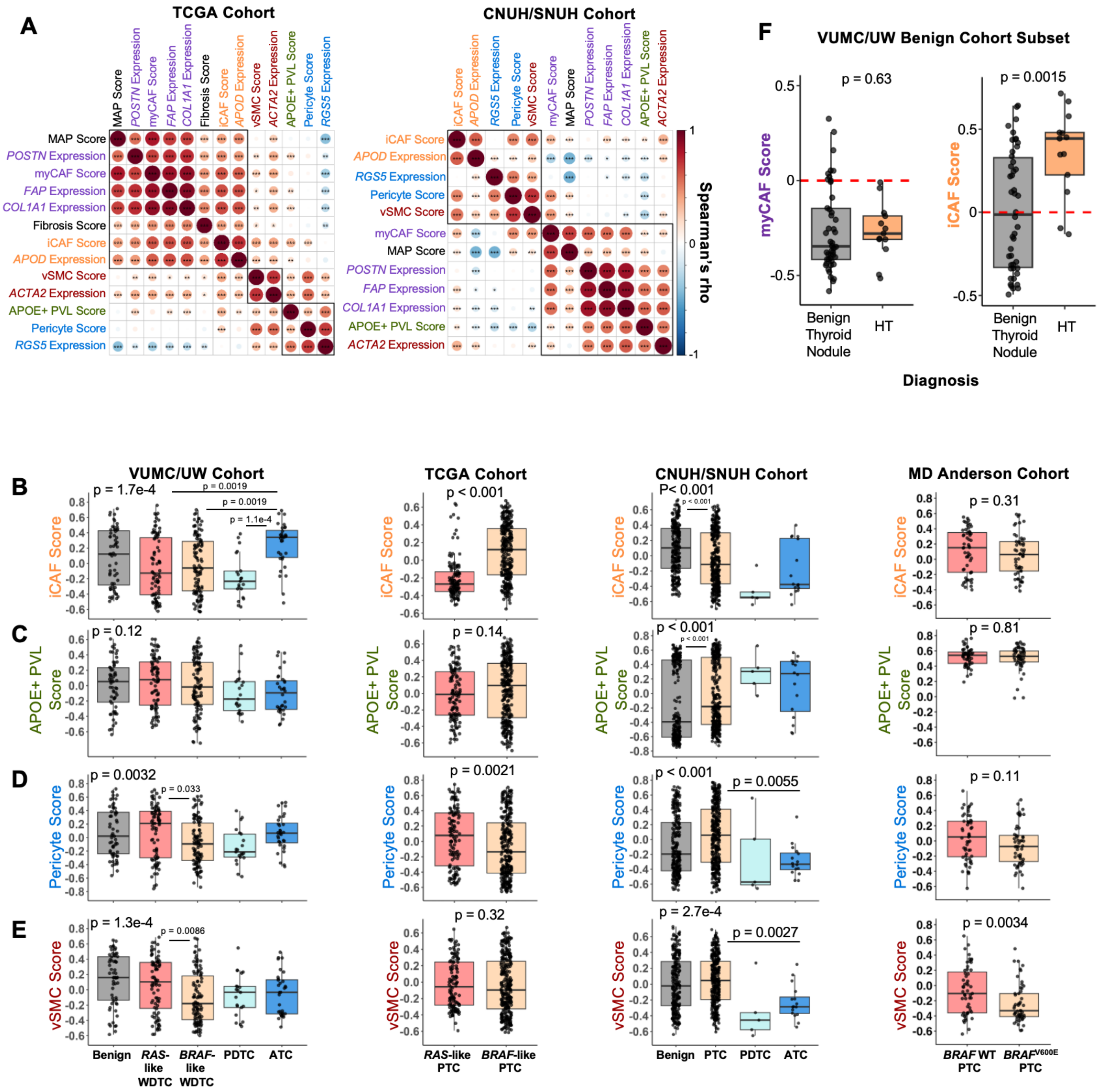

Supplemental Figure 4: Stromal cell abundance in bulk RNA-sequencing cohorts. (**A**) Corrplot showing Spearman's rho correlations between single-sample gene set variation analysis (ssGSVA) scores for stromal subpopulations, ssGSVA score for molecular aggression and prediction (MAP) score, and expression of marker genes for stromal subpopulations in The Cancer Genome Atlas PTC cohort (TCGA cohort, left) and Chungnam/Seoul National University Hospitals cohort (CNUH/SNUH cohort, right). Axes ordered by hierarchical clustering. Boxes indicate hierarchical clustering groups. Significance levels indicate *p<0.05, **p<0.01, or ***p<0.001. (**B**-**E**) Boxplots showing ssGSVA scores for (**B**) inflammatory cancer-associated fibroblasts (iCAFs), (**C**) APOE+ perivascular-like cells (APOE+ PVL), (**D**) pericytes, and (**E**) vascular smooth muscle cells (vSMCs) by diagnosis across four distinct bulk RNA-sequencing cohorts. (**F**) Boxplots showing myCAF ssGSVA scores (left) and iCAF ssGSVA scores (right) for benign thyroids split by diagnosis of benign thyroid nodules or Hashimoto thyroiditis (HT). P-values for **B-F** calculated with Wilcoxon rank-sum test. Kruskal-Wallis test with subsequent pairwise Wilcoxon rank-sum tests with Bonferroni correction was used when comparing more than two groups. Abbreviations: myCAF, myofibroblast cancer-associated fibroblast; PTC, papillary thyroid cancer.

Supplemental Figure 5: *BRAF* WT ATCs have heterogeneous expression of mesenchymal markers.

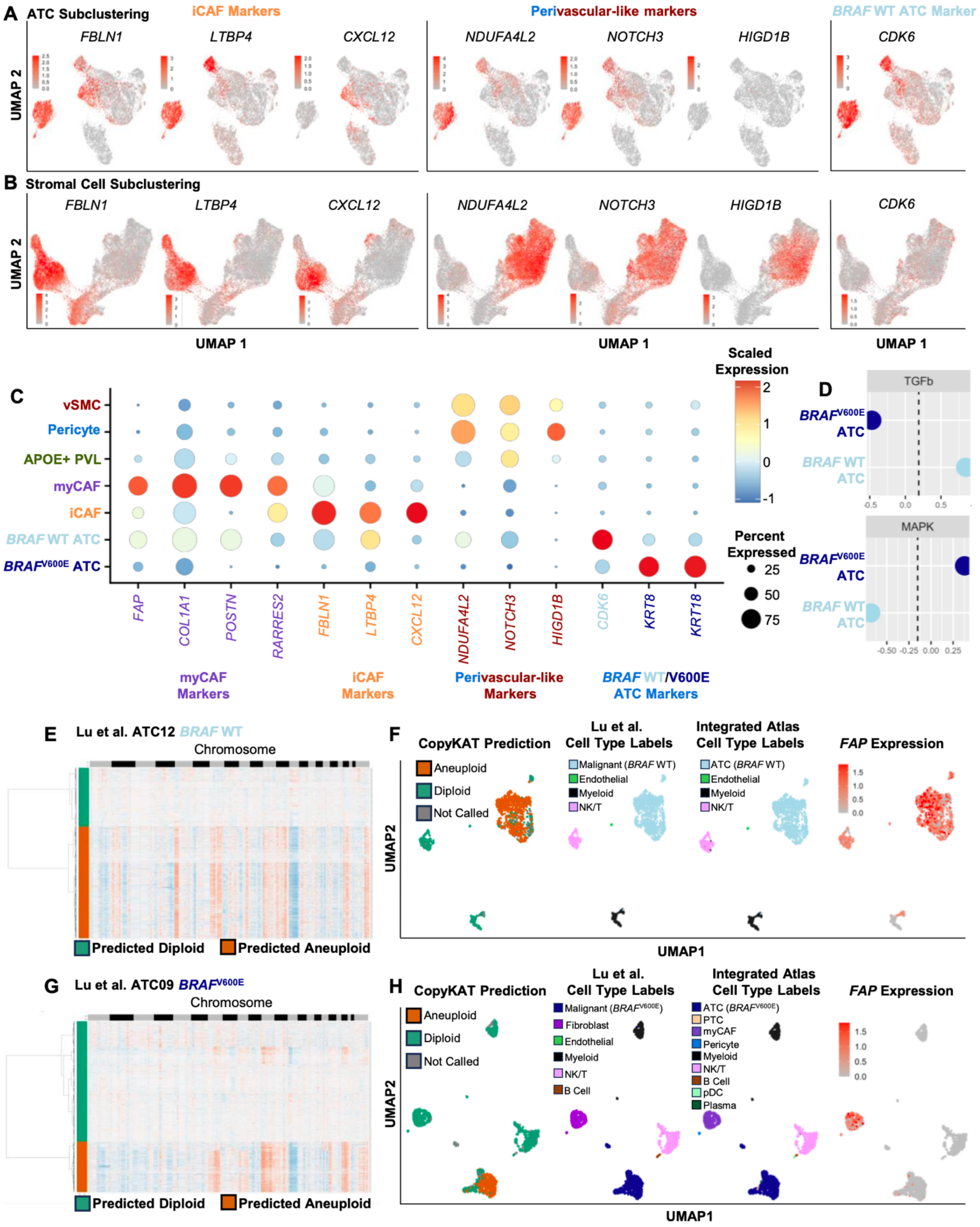

129

Supplemental Figure 5: *BRAF* WT ATCs have heterogeneous expression of mesenchymal markers. (**A** and **B**) Uniform Manifold Approximation and Projection (UMAP) plots of (**A**) anaplastic thyroid carcinoma (ATC) tumor cell subclustering and (**B**) stromal cell subclustering colored by expression of inflammatory cancer-associated fibroblast (iCAF) markers *FBLN1*, *LTBP4*, *CXCL12* (left), perivascular-like (PVL) markers *NDUFA4L2*, *NOTCH3*, *HIGD1B* (middle), and the ATC marker *CDK6* (right). (**C**) Dot plot showing scaled expression of myofibroblast CAF (myCAF), iCAF, PVL, and ATC marker genes for stromal cell subpopulations (iCAF; myCAF; APOE+ PVL; pericyte; vascular smooth muscle cell, vSMC), *BRAF* wild type (WT) ATC tumor cells, and *BRAF*^{V600E} ATC tumor cells. (**D**) PROGENy signaling pathway activity scores for *BRAF* WT and *BRAF*^{V600E} ATC cells. (**E**) Heatmap showing gene expression (orange = high; blue = low) across chromosomal location (x-axis) for all cells (y-axis, cells in order of hierarchical clustering) from a representative *BRAF* WT ATC (ATC12 from Lu et al.) with CopyKAT aneuploid prediction shown (green = diploid; orange = aneuploid). (**F**) UMAP of independent analysis of single-cell RNA-sequencing from ATC12 from Lu et al. colored from left to right by CopyKAT prediction, cell type labels provided by Lu et al., cell type labels from integrated thyroid cancer atlas, and *FAP* expression. (**G**) Heatmap showing gene expression (orange = high; blue = low) across chromosomal location (x-axis) for all cells (y-axis, cells in order of hierarchical clustering) from a representative *BRAF*^{V600E} ATC (ATC09 from Lu et al.) with CopyKAT aneuploid prediction shown (green = diploid; orange = aneuploid). (**H**) UMAP of independent analysis of single-cell RNA-sequencing from ATC09 from Lu et al. colored from left to right by CopyKAT prediction, cell type labels provided by Lu et al., cell type labels from integrated thyroid cancer atlas, and *FAP* expression.

Supplemental Figure 6: myCAFs do not correlate with patient survival in ATC.

A

Wald Statistics for `Surv(PFS.from.ini.therapy.complete, event.PFS)`

	χ^2	d.f.	*P*
group (Factor+Higher Order Factors)	49.70	2	<0.0001
All Interactions	5.20	1	0.0226
myCAF_Score (Factor+Higher Order Factors)	5.25	2	0.0726
All Interactions	5.20	1	0.0226
group × myCAF_Score (Factor+Higher Order Factors)	5.20	1	0.0226
**TOTAL**	65.80	3	<0.0001

B

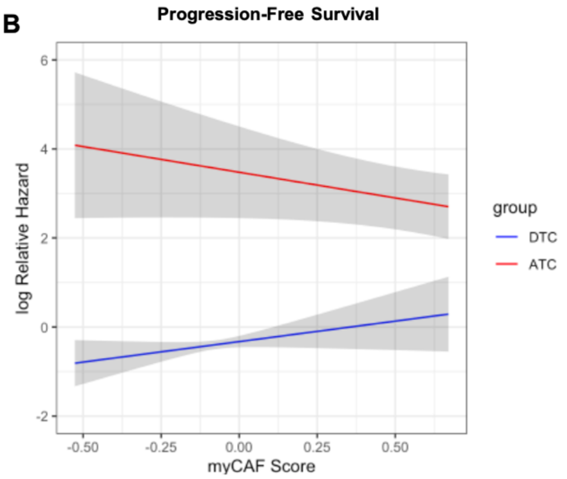

C

Wald Statistics for `Surv(OS.from.ini.therapy.complete, event.OS)`

	χ^2	d.f.	*P*
group (Factor+Higher Order Factors)	46.05	2	<0.0001
All Interactions	5.05	1	0.0246
myCAF_Score (Factor+Higher Order Factors)	6.15	2	0.0461
All Interactions	5.05	1	0.0246
group × myCAF_Score (Factor+Higher Order Factors)	5.05	1	0.0246
**TOTAL**	58.35	3	<0.0001

D

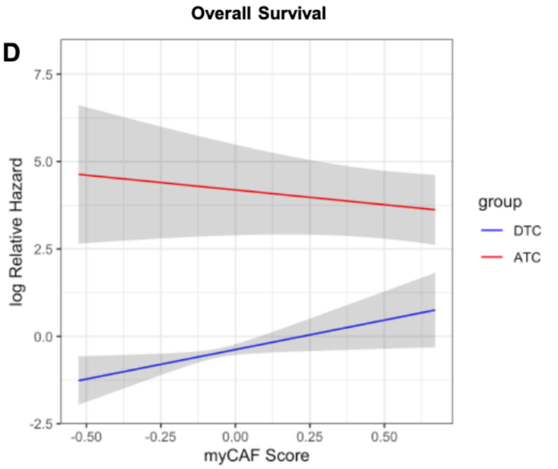

E

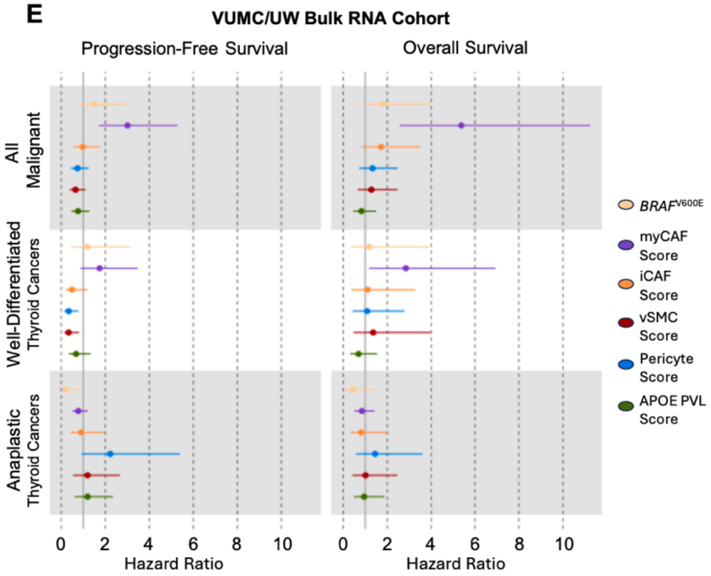

Supplemental Figure 6: myCAFs do not correlate with patient survival in ATC. (**A** and **B**) Multivariable cox regression for progression-free survival (PFS) with an interaction term of diagnostic group (differentiated thyroid cancer, DTC or anaplastic thyroid cancer, ATC) by continuous myofibroblast cancer-associated fibroblast (myCAF) score with (**A**) showing Wald Statistics and (**B**) showing the partial effect plot. (**C** and **D**) Multivariable cox regression for overall survival (OS) with an interaction term of diagnostic group (DTC or ATC) by continuous myCAF score with (**C**) showing Wald Statistics and (**D**) showing the partial effect plot. (**E**) PFS (left) and OS (right) forest plots with interquartile range hazard ratios and 95% confidence intervals for the VUMC/UW bulk RNA-sequencing cohort split into malignant samples (top), well-differentiated thyroid cancers (middle), or ATCs (bottom). Hazard ratios are shown for *BRAF*^{V600E} mutation and stromal cell ssGSVA scores. Abbreviations: iCAF, inflammatory CAF; vSMC, vascular smooth muscle cell; PVL, perivascular-like.

Supplemental Figure 7: Thyroid cancer spatial sequencing atlas.

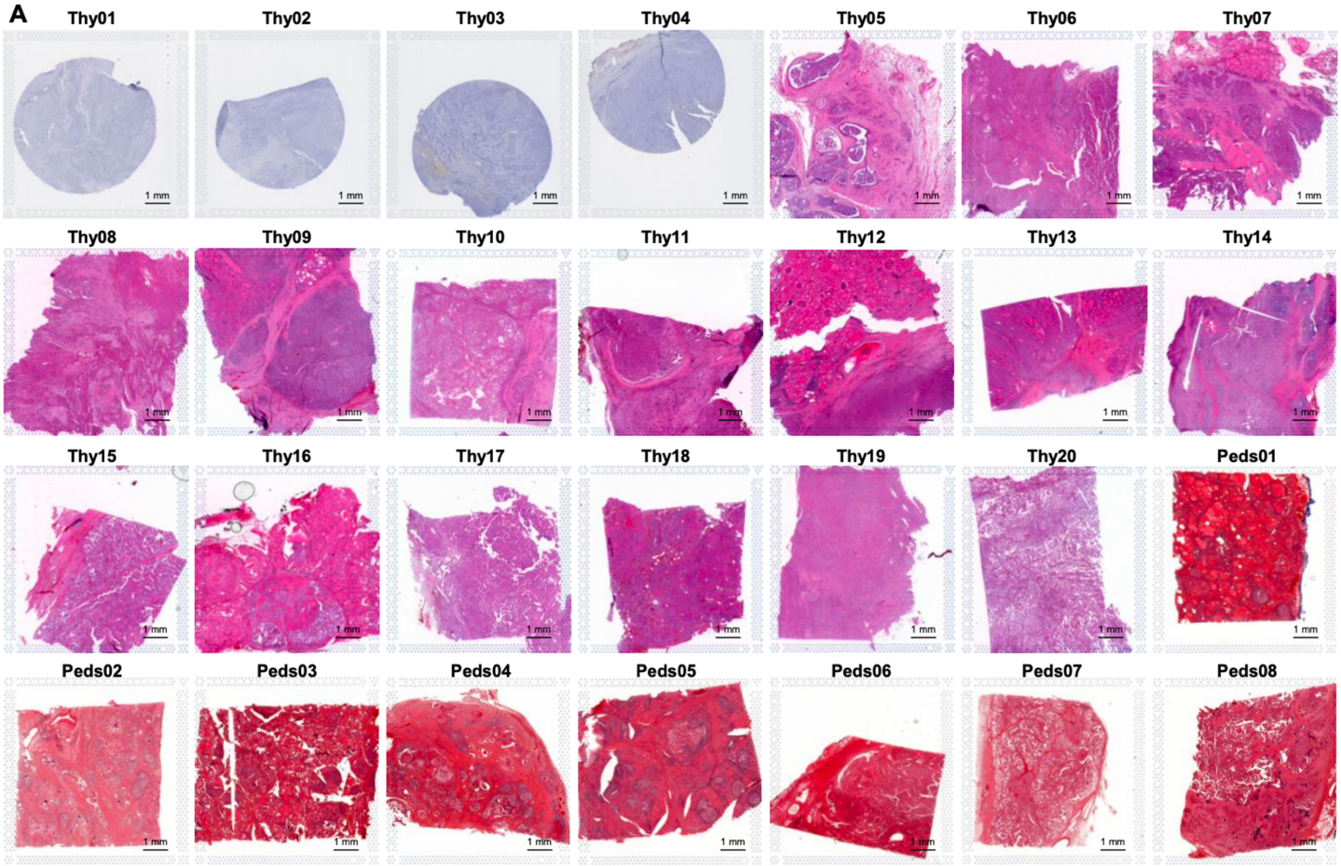

B

Sample	Age	Histology	$BRAF^{V600E}$	*RET* Fusion
Thy01	Adult	PTC/ATC	YES	NO
Thy02	Adult	ATC	NO	NO
Thy03	Adult	ATC	NO	NO
Thy04*	Adult	ATC	YES	NO
Thy05	Adult	PTC/ATC	YES	NO
Thy06	Adult	PTC/ATC	YES	NO
Thy07	Adult	PTC	YES	NO
Thy08	Adult	ATC	YES	NO
Thy09	Adult	PTC/ATC	NO	NO
Thy10	Adult	PTC/ATC	YES	NO
Thy11*	Adult	PTC/ATC	YES	NO
Thy12	Adult	ATC	YES	NO
Thy13	Adult	PTC/ATC	NO	NO
Thy14	Adult	ATC	NO	NO
Thy15	Adult	PTC	YES	NO
Thy16	Adult	PTC	YES	NO
Thy17	Adult	PTC	YES	NO
Thy18	Adult	PTC	YES	NO
Thy19	Adult	ATC	NO	NO
Thy20	Adult	ATC	NO	NO
Peds01	Pediatric	PTC	NO	YES
Peds02	Pediatric	PTC	NO	YES
Peds03	Pediatric	PTC	NO	YES
Peds04	Pediatric	PTC	NO	YES
Peds05	Pediatric	PTC^	NO	YES
Peds06	Pediatric	PTC	NO	YES
Peds07	Pediatric	PTC	NO	YES
Peds08	Pediatric	PTC	NO	YES

*Indicates distinct samples from the same tumor
^Indicates background Hashimoto thyroiditis

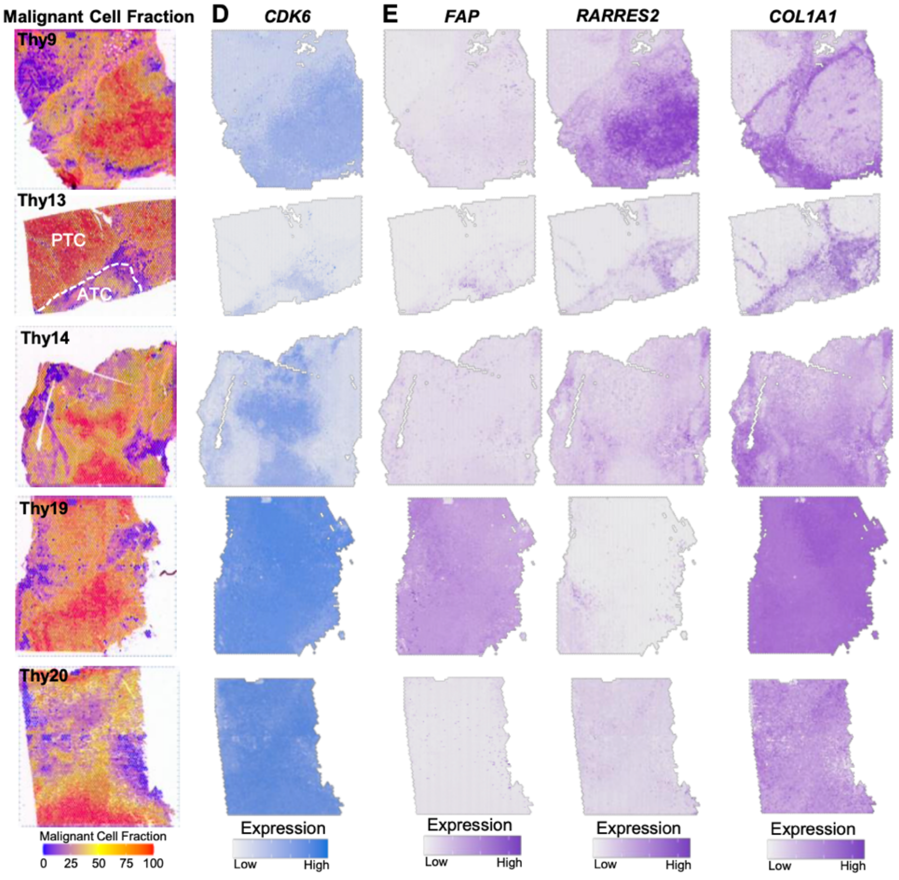

Supplemental Figure 7: Thyroid cancer spatial sequencing atlas. (**A**) Hematoxylin and eosin staining of 28 spatial transcriptomics tumor samples. (**B**) Table with age, histologic subtype, and mutation data for 28 spatial transcriptomics tumor samples. *Indicates distinct samples from the same tumor. ^Indicates background Hashimoto thyroiditis. (**C**) Malignant cell fraction of spatial transcriptomics barcodes for five *BRAF* wild type (WT) anaplastic thyroid carcinoma (ATC) tumors calculated by Spatial Cellular Estimator for Tumors (SpaCET) (75). (**D**) Expression of ATC marker *CDK6* across five *BRAF* WT ATC spatial transcriptomic samples. (**E**) Expression of myCAF markers *FAP*, *RARRES2*, *COL1A1* across five *BRAF* WT ATC spatial transcriptomic samples.

Supplemental Figure 8: IHC of iCAF and myCAF markers in benign and malignant thyroid lesions.

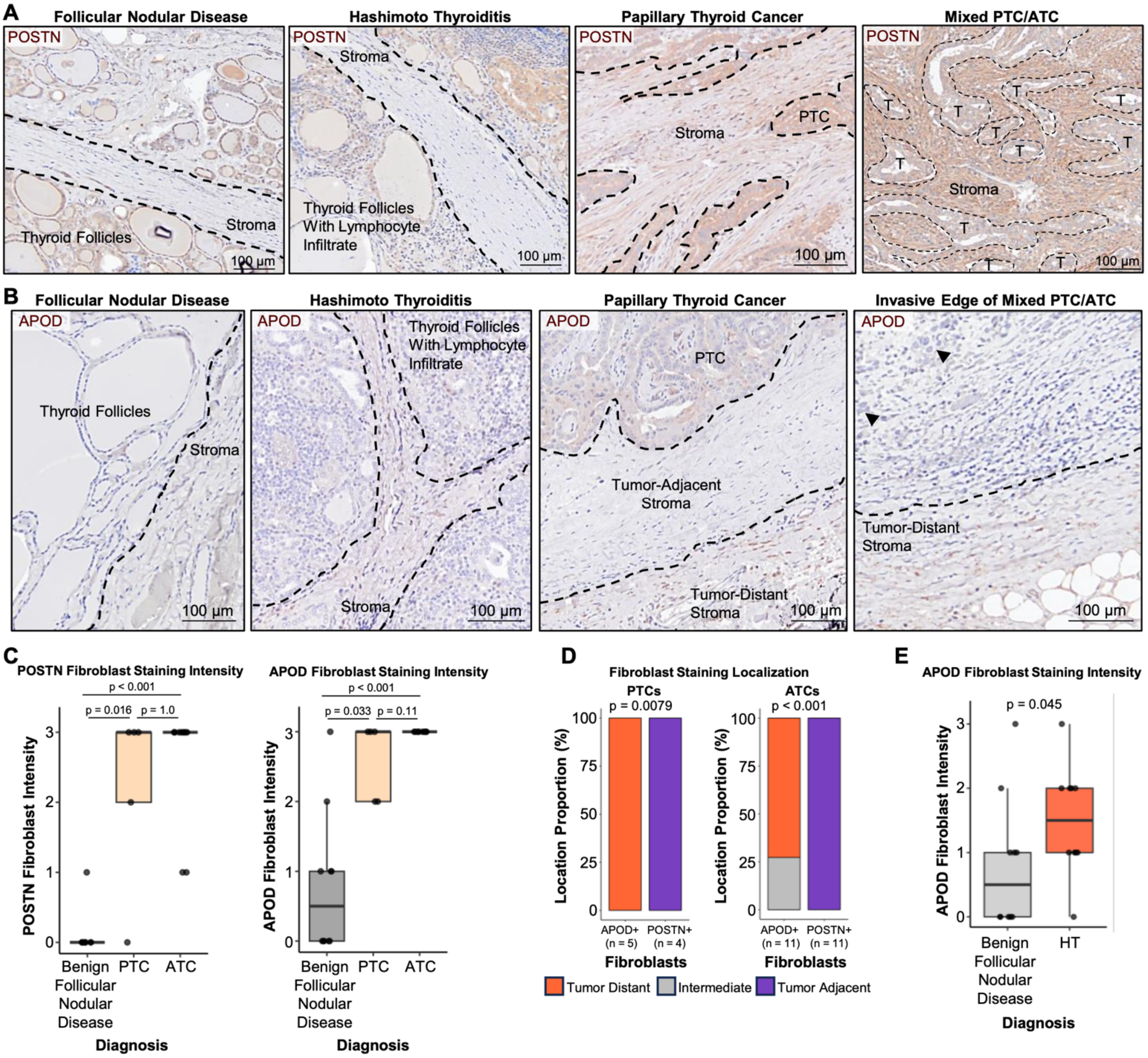

170

Supplemental Figure 8. IHC of iCAF and myCAF markers in benign and malignant thyroid lesions. (**A**) Representative periostin (POSTN) IHC images from staining of 10 thyroids with benign follicular nodular disease, 10 thyroids with Hashimoto thyroiditis (HT), five papillary thyroid carcinomas (PTCs), six mixed papillary/anaplastic thyroid cancer samples, and five anaplastic thyroid carcinomas (ATCs). (**B**) Representative ApolipoproteinD (APOD) IHC images from staining of 10 thyroids with follicular nodular disease, 10 thyroids with HT, 5 PTCs, 6 PTC/ATC samples, and 5 ATCs. Black arrowheads point to tumor cells. (**C**) Boxplots showing pathologist quantification of staining intensity on stromal cells for POSTN (left) and APOD (left) by sample histology. P-values calculated with pairwise Wilcoxon rank-sum tests with Bonferroni correction. (**D**) Bar plots showing pathologist scoring of fibroblast localization relative to tumor cells in PTCs (left) and ATCs (right) for fibroblasts staining with APOD and POSTN. P-values calculated with Fisher's exact test. (**E**) Boxplot showing pathologist quantification of APOD staining intensity on stromal cells from follicular nodular disease (left) and HT (right). P-value calculated with one-sided Wilcoxon rank-sum test.

Supplemental Figure 9: Detection of iCAFs in Hashimoto thyroiditis.

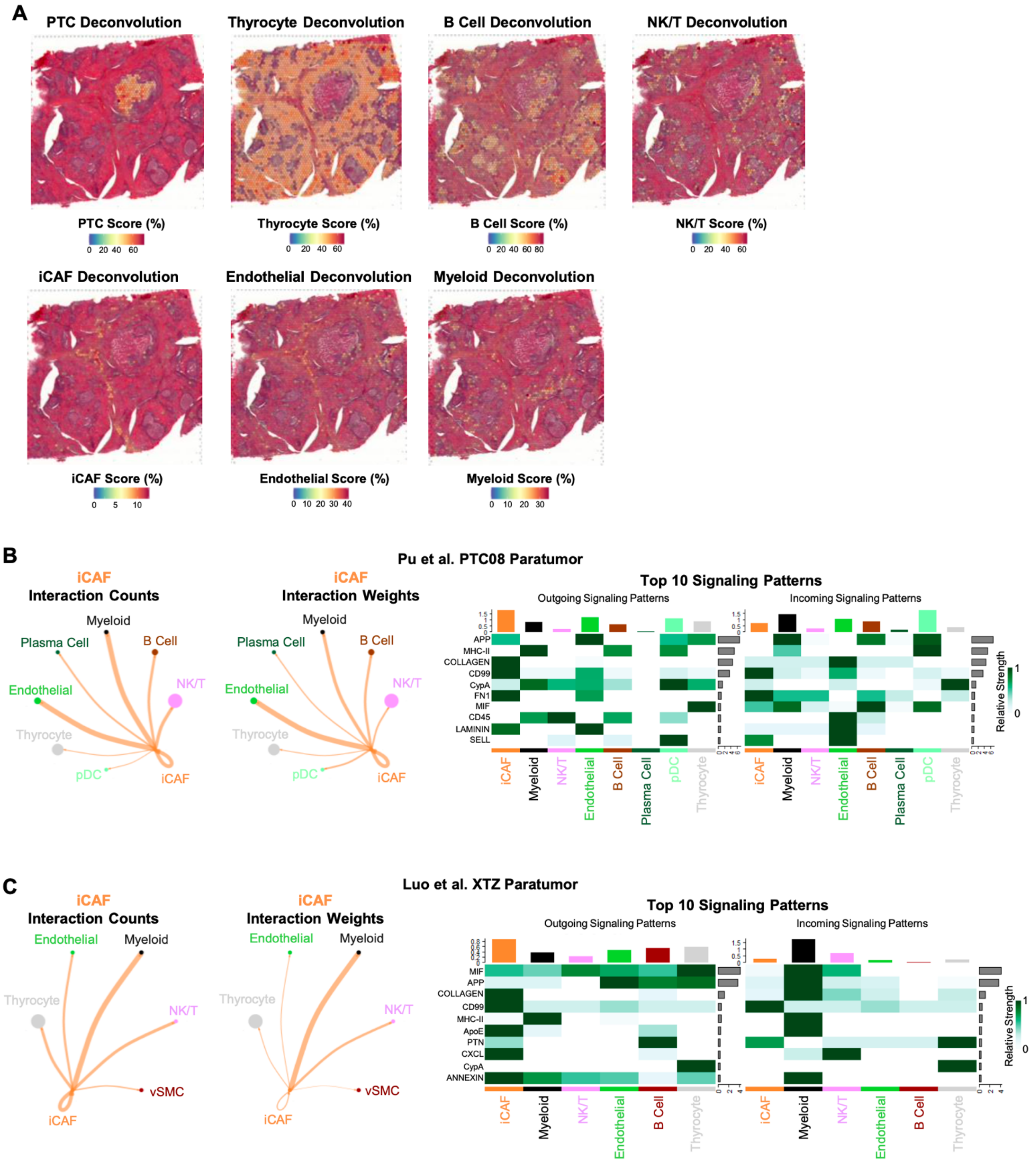

Supplemental Figure 9. Detection of iCAFs in Hashimoto thyroiditis. (**A**) Spatial feature plots showing Robust Cell Type Decomposition (RCTD) of a pediatric papillary thyroid cancer (PTC) sample with background Hashimoto thyroiditis (Peds05). Top row from left to right: PTC RCTD, thyrocyte RCTD, B cell RCTD, and Natural Killer/T cell (NKT) RCTD. Bottom row from left to right: inflammatory cancer-associated fibroblast (iCAF) RCTD, endothelial RCTD, and myeloid RCTD. (**B** and **C**) Ligand-receptor interaction analysis in paratumor single-cell samples (**B**) Pu et al. PTC08 paratumor and (**C**) Luo et al. XTZ paratumor that have background Hashimoto thyroiditis and iCAF populations. The width of lines indicates either the interaction count or the interaction weight of iCAFs signaling to other stromal populations. Right: the top 10 signaling pathways with outgoing signal scores on the left and incoming signal scores on the right. Single-cell populations are labeled on the bottom.

Supplemental Figure 10: Spatial deconvolution of stromal populations.

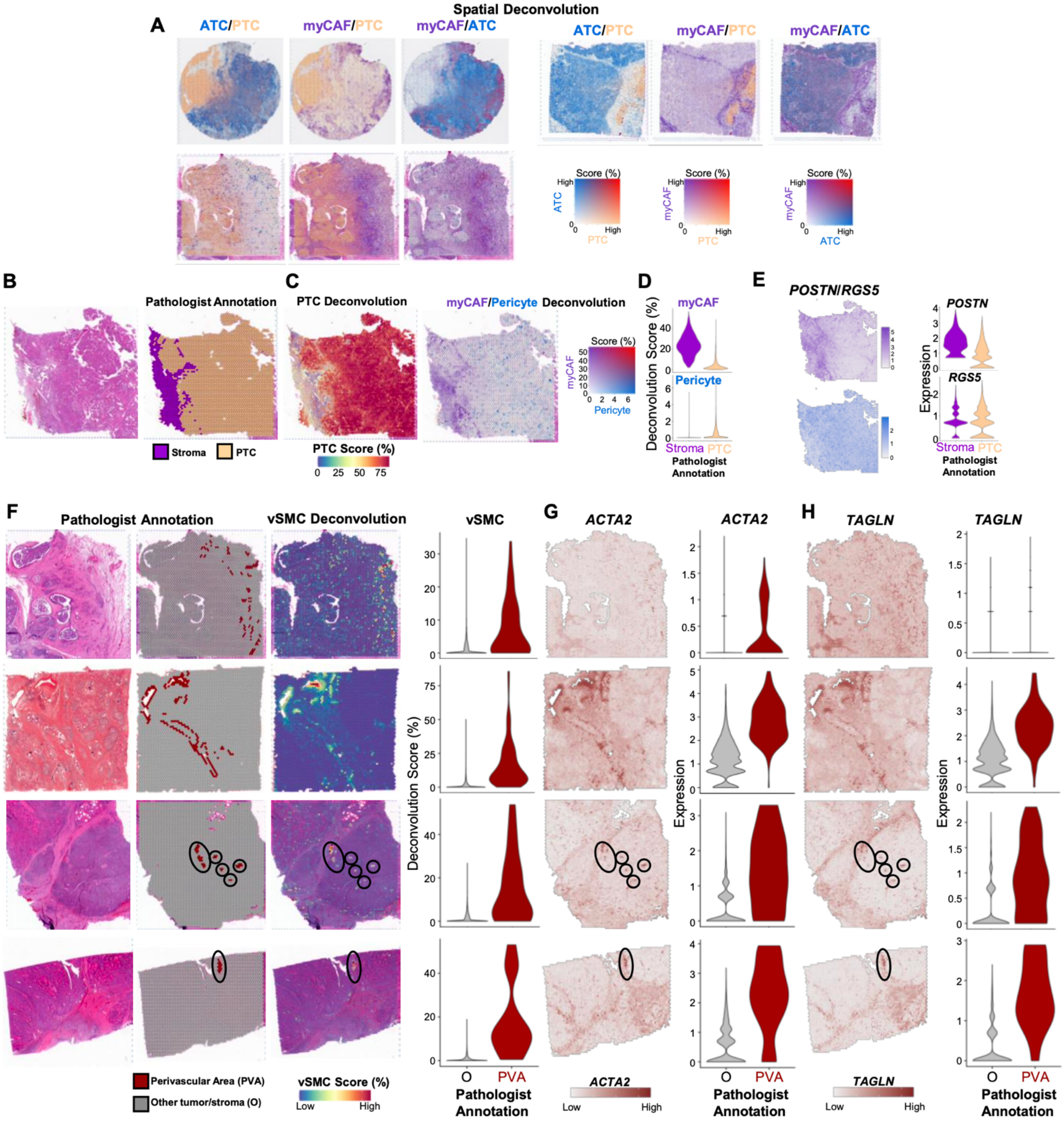

198

Supplemental Figure 10: Spatial deconvolution of stromal populations. (**A**) Spatial feature plots of three mixed papillary and anaplastic thyroid cancer (PTC/ATC) samples (Thy1 Thy5, Thy10) showing ATC (blue), PTC (light orange), and myofibroblast cancer-associated fibroblast (myCAF, purple) RCTD scores. Mixing of deconvoluted populations is shown by a color gradient that becomes red. (**B**) Hematoxylin and eosin staining of representative PTC Thy17 (left) with pathologist annotation of PTC (light orange) and stromal (purple) spatial barcodes (right). (**C**) Spatial feature plots of representative PTC Thy17 showing Robust Cell Deconvolution (RCTD) of PTC (left, red color gradient) and myCAF/pericyte (right, purple/blue color gradients) populations. (**D**) Violin plots showing myCAF RCTD and pericyte RCTD scores within pathologist annotated stromal (purple) and PTC (light orange) barcodes. (**E**) Spatial feature plots (left) and violin plots (right) depicting myCAF (*POSTN*) and pericyte (*RGS5*) marker gene expression in pathologist annotated stromal and PTC regions. (**F**) Pathologist spatial annotation of large perivascular areas (PVAs) and spatial feature plot of vascular smooth muscle cell (vSMC) RCTD scores across four tumors. Left: hematoxylin and eosin staining and pathologist annotations. Middle: vSMC RCTD score plots. Right: violin plots of vSMC RCTD score stratified by barcodes whether spatial barcodes are in large PVAs. (**G** and **H**) Spatial feature plots (left) and associated violin plots (right) for vSMC marker genes (**G**) *ACTA2* and (**H**) *TAGLN*. From top to bottom tumors are Thy5, Peds02, Thy9, Thy13 for **F-H**.

Supplemental Figure 11: Ligand-receptor interaction analysis between tumor and stromal populations.

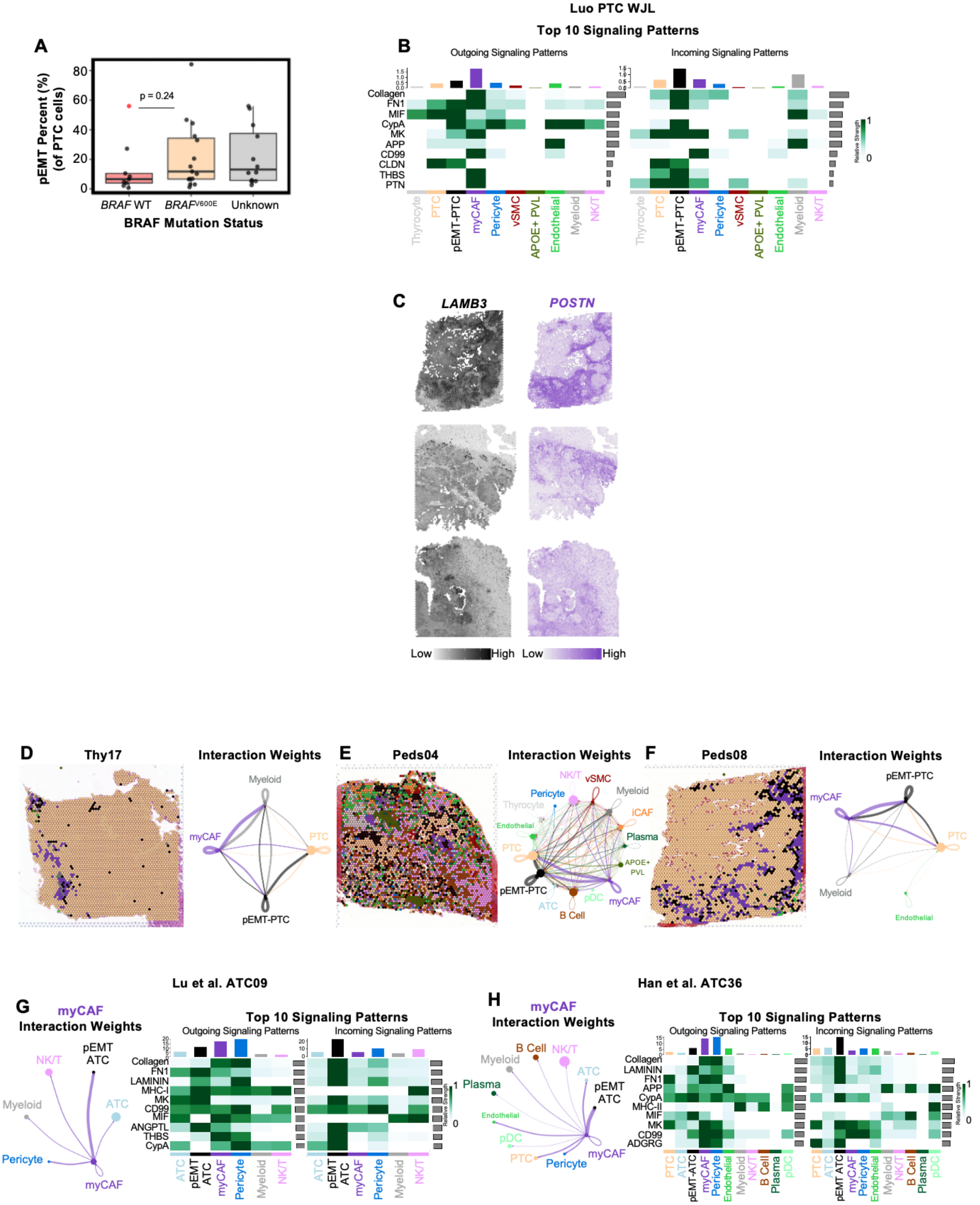

216

Supplemental Figure 11: Ligand-receptor interaction analysis between tumor and stromal populations. (**A**) Boxplot depicting the percent of partial epithelial-mesenchymal-transition (pEMT) cells in the broad papillary thyroid cancer (PTC) cluster for each single-cell sample with at least 100 PTC cells split by *BRAF* wild-type (WT, light red), *BRAF*^{V600E} (light orange), or unknown *BRAF* status. P-value between *BRAF* WT and *BRAF*^{V600E} calculated with Wilcoxon rank-sum test. The red colored dot indicates a paratumor sample. (**B**) Heatmap showing the top 10 signaling patterns in representative PTC WJL from Luo et al. with outgoing signal scores on the left and incoming signal scores on the right. Single-cell populations are labeled on the bottom. (**C**) Spatial feature plots of pEMT-PTC (*LAMB3*, black) and myCAF (*POSTN*, purple) marker gene expression. Samples shown are a pediatric PTC (Peds08, top), adult PTC (Thy7, middle), and adult mixed PTC/ATC (Thy5, bottom). (**D-F**) Spatial ligand-receptor interaction analysis at the invasive edge of PTC samples for (**D**) Thy17, (**E**) Peds04, and (**F**) Peds08. Left: Spatial plot with labeling of each spatial barcodes by the population with the highest Robust Cell Type Decomposition (RCTD) score in each sample. Right: spatial ligand-receptor interaction weights between labeled populations on the left. The width of lines indicates interaction weights. (**G** and **H**) Ligand-receptor interaction analysis in *BRAF*^{V600E} anaplastic thyroid cancer (ATC) single-cell samples (**G**) Lu et al. ATC09 and (**H**) Han et al. ATC36 that have pEMT-ATC populations. Left: ligand-receptor interaction weights between single-cell populations. The width of lines indicates interaction weights. Right: the top 10 signaling patterns with outgoing signal scores on the left and incoming signal scores on the right. Single-cell populations are labeled on the bottom. Abbreviations: myCAF, myofibroblast cancer-associated fibroblast; NK/T; vSMC, vascular smooth muscle cell; PVL, perivascular-like; natural killer/T cell.

Supplemental Figure 12: pEMT gene signature is associated with aggressive thyroid cancer tumor phenotypes.

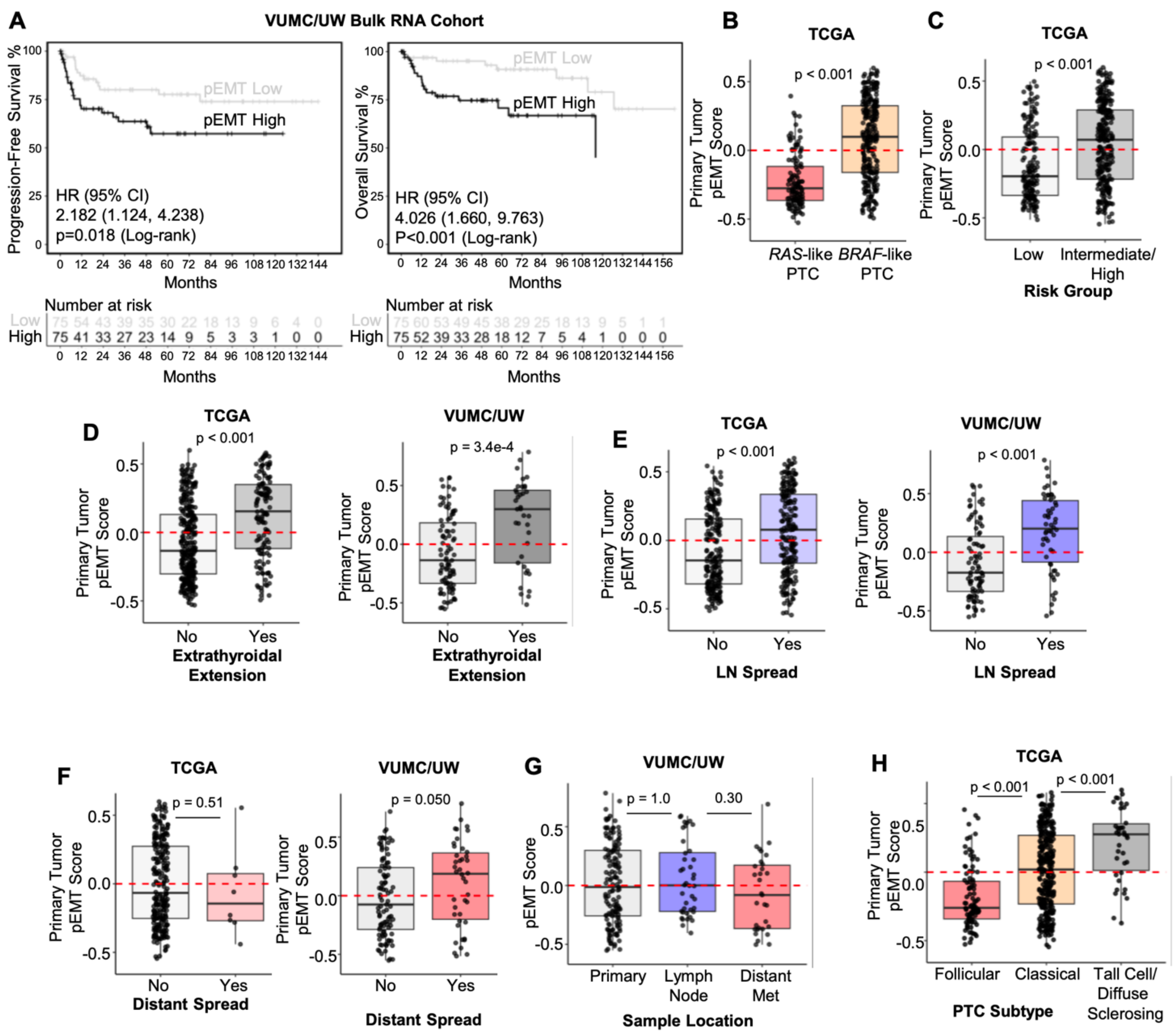

237

Supplemental Figure 12: pEMT gene signature is associated with aggressive thyroid cancer tumor phenotypes. (**A**) Progression-free survival (left) and overall survival (right) Kaplan-Meier curves for the Vanderbilt University Medical Center/University of Washington thyroid cancer bulk RNA-sequencing cohort (VUMC/UW bulk RNA cohort) for single-sample gene set variation analysis (ssGSVA) of a partial epithelial-mesenchymal transition (pEMT) gene list from Puram et al. split into pEMT-high and pEMT-low by 50th percentile pEMT score. P-values calculated with log-rank test. (**B-F**) Boxplots depicting primary tumor pEMT ssGSVA score in (**B**) The Cancer Genome Atlas (TCGA) papillary thyroid carcinomas (PTCs) stratified by *RAS*-like versus *BRAF*-like gene expression, (**C**) TCGA PTCs stratified by risk group as defined by the 2009 American Thyroid Association guidelines, (**D**) TCGA PTCs (left) and VUMC/UW malignant cohort (right) stratified by the presence or absence of extrathyroidal extension, (**E**) TCGA PTCs (left) and VUMC/UW malignant cohort (right) stratified by the presence or absence of lymph node spread, and (**F**), TCGA PTCs (left) and VUMC/UW malignant cohort (right) stratified by the presence or absence of distant metastasis. P-values for **B-F** calculated with Wilcoxon rank-sum test. (**G**) pEMT score in the VUMC/UW malignant cohort stratified by sample location. (**H**) Primary tumor pEMT score for TCGA PTCs stratified by histology subtype. Tall cell and diffuse sclerosing PTC subtypes are grouped. P-values for **G** and **H** calculated with pairwise Wilcoxon rank-sum tests with Bonferroni correction.
